# Task‐induced brain functional connectivity as a representation of schema for mediating unsupervised and supervised learning dynamics in language acquisition

**DOI:** 10.1002/brb3.2157

**Published:** 2021-05-05

**Authors:** Hiroyuki Akama, Yixin Yuan, Shunji Awazu

**Affiliations:** ^1^ Institute of Liberal Arts/Department of Life Science and Technology Tokyo Institute of Technology Tokyo Japan; ^2^ Marcus Autism Center Children’s Healthcare of Atlanta Atlanta GA USA; ^3^ Division of Autism & Related Disabilities Department of Pediatrics Emory University School of Medicine Atlanta GA USA; ^4^ Faculty of Humanities and Social Sciences Jissen Women’s University Tokyo Japan

**Keywords:** artificial language grammar, default mode network, functional connectivity, language learning, schema theory, supervised learning, unsupervised learning

## Abstract

**Introduction:**

Based on the schema theory advanced by Rumelhart and Norman, we shed light on the individual variability in brain dynamics induced by hybridization of learning methodologies, particularly alternating unsupervised learning and supervised learning in language acquisition. The concept of “schema” implies a latent knowledge structure that a learner holds and updates as intrinsic to his or her cognitive space for guiding the processing of newly arriving information.

**Methods:**

We replicated the cognitive experiment of Onnis and Thiessen on implicit statistical learning ability in language acquisition but included additional factors of prosodic variables and explicit supervised learning. Functional magnetic resonance imaging was performed to identify the functional network connections for schema updating by alternately using unsupervised and supervised artificial grammar learning tasks to segment potential words.

**Results:**

Regardless of the quality of task performance, the default mode network represented the first stage of spontaneous unsupervised learning, and the wrap‐up accomplishment for successful subjects of the whole hybrid learning in concurrence with the task‐related auditory language networks. Furthermore, subjects who could easily “tune” the schema for recording a high task precision rate resorted even at an early stage to a self‐supervised learning, or “superlearning,” as a set of different learning mechanisms that act in synergy to trigger widespread neuro‐transformation with a focus on the cerebellum.

**Conclusions:**

Investigation of the brain dynamics revealed by functional connectivity imaging analysis was able to differentiate the synchronized neural responses with respect to learning methods and the order effect that affects hybrid learning.

## Graphical abstract

Based on the schema theory advanced by Rumelhart and Norman, we shed light on the individual variability in brain dynamics induced by hybridization of learning methodologies, particularly alternating unsupervised learning and supervised learning in language acquisition. Functional magnetic resonance imaging was performed to identify the functional network connections for schema updating by alternately using unsupervised and supervised artificial grammar learning tasks to segment potential words. The default mode network represented the first stage of spontaneous unsupervised learning, and the wrap‐up accomplishment for successful subjects of the whole hybrid learning in concurrence with the task‐related auditory language networks.

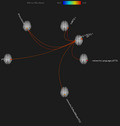

## 1 INTRODUCTION

So‐called unsupervised learning (USL) implies spontaneous grasping efforts to acquire knowledge and skills (Clark, 2001), whereas supervised learning (SL) refers to accepting the guidance of a teacher to improve levels of ability in target domains (Fagg, 1994). The relationships between USL and SL have been defined according to the theory of “schema” (Bartlett, 1932; Schank & Abelson, 1977), which has been previously defined as “active, interrelated knowledge structures, actively engaged in the comprehension of arriving information, guiding the execution of processing operations” (Rumelhart & Norman, 1976). The schema theory was initially applied to the field of learning motor skills (Salmoni et al., 1984; Sherwood & Lee, 2004). The term was later broadened to accommodate an artificial intelligence‐oriented perspective: “a *schema* is what is learned about some aspect of the world, combining knowledge with their corresponding application processes; a *schema instance* is an active deployment of these processes” (Arbib, 1992). To date, the schema theory has been further diversely conceptualized across various cognitive domains.

The schema theory can similarly be applied to characterize verbal learning (Carrel & Eisterhold, 1983), although its implementation in the language learning literature has been largely limited to the domain of reading or listening comprehension (Al‐Issa, 2006; Long, 1989). Extending the schema theory to speech acquisition paradigms would allow for the better understanding of innate language development and subsequently facilitate targeted language training. For example, the canonical babbling of infants recruits both procedural and declarative learning processes, which can be further elucidated as the cooperation between USL and SL. The babbling process is initially predominated by USL, where an initial schema is formed and guides the implicit recognition of auditory patterns that were previously encountered. SL then begins to contribute at around 6 months of age. Selective attention emerges at this age, allowing for direct comparisons between explicit mental storage of auditory patterns and external canonical feedback that in turn facilitates schema update and development (Vihman, 2017). On the other hand, even though SL is a typical model of successful second‐language learning at school (Lyster et al., 2013), the learning of canonical categories models (Gogate et al., 2006; Goudbeek et al., 2009) will be somewhat influenced by the “schema” pre‐established from the speaker's native language acquisition, making it subject to a mixed model that includes both SL and USL. This is because, through implicitly learning the prosodic cues (Brodsky et al., 2007; Da Silva, 2015; Johnson & Heidl, 2009) and transitional probabilities between patterns for word identification (Pelucchi et al., 2009; Saffran et al., 1996; Xie, 2012), infants not only acquire their native tongue but also establish a lasting “schema” that continues to be influential in later learning of a second language (Cutler et al., 1992; Finn, Hudson, & Kam, 2008; Otake et al., 1993; Rodriguez‐Fornells et al., 2005; Spivey & Marian, 1999; Weber & Cutler, 2006). These examples show that both first‐ and second‐language learning fall under a form of “self‐supervised learning” with interactive processing and organization of knowledge and can therefore be effectively modeled via the schema theory (Kröger & Bekolay, 2019).

A latent “schema” is the medium responsible for creating learning dynamics by relaying active spontaneous formation and passive reception and thus mediates between USL and SL (Kröger & Bekolay, 2019; Selinker, 1972; Vihman, 2017). According to Rumelhart and Norman (1976), a schema can be updated according to three modes of learning: “accretion,” “tuning,” and “restructuring.” When incoming information coincides with the agent's original intrinsic schema, that particular experience will be engendered through USL and added to the pre‐established cognitive space through the process of “accretion.” However, when discrepancy arises between new information and the original schema, the agent actively employs SL to update the latter, either through “tuning” variables to make minor structural changes when conflicts are mild or through large‐scale “restructuring” events where a completely new schema is developed to account for the incoming information.

Note that the modulation of the schema can be reformulated and computationally implemented through the connectionist model based on the error‐based learning theory (Cleeremans & McClelland, 1991; Liu & Liu, 2014). More specifically, we presume that updating the schema would be initiated in each learner by a cycle of prediction, error detection, and subsequent readjustment of the learning function. This learning process is analogous to back propagation in the middle (hidden) layers of an artificial neural network. By repeating the learning steps, the weights given to the middle (hidden) layers are sequentially renewed such that the residuals between the output and teacher signals to be learned are minimized and converge toward stability. This process of back propagation, a key defining step in machine learning, might be algorithmically similar to that of underlying mechanisms of schema update to account for learned new experiences. We therefore hypothesize that this schema‐update mechanism is physically implemented in the human brain.

However, there is limited literature on the neural mechanism that creates such dynamic schema regulation processes in language learning. A number of clues have been found that might eventually elucidate the neural underpinnings of USL, SL, and their intermediating schema. Previous studies have demonstrated that the neural systems underpinning nonmodality‐specific learning involve the basal ganglia, cerebellum, and limbic system (Caligiore et al., 2019; Hertrich et al., 2016). Referencing the functional network connecting these areas in the brain, Caligiore et al. (2019) formulated a hypothesis of “superlearning” as an integration of SL, USL, and reinforcement learning. His hypothesis elaborated a model based on the ideas of Schweighofer et al. (2004) to explain how USL could strengthen SL via an information transfer mechanism. However, the dynamism of the mutual relationship between the various types of learning is hitherto unexplored from the perspective of individual variability in modulation of the functional network.

The neural implementation of schema update is further complicated by previous study designs that are longitudinal and sparsely sampled in nature, where data from two or more functional magnetic resonance imaging (fMRI) sessions were obtained for subjects over the course of several months (Bubbico et al., 2019; Chai et al., 2016; Saidi et al., 2013; Vinals, 2016). Although such experimental designs allow for the detection of long‐term changes induced by learning—for example, for a change in association weights of the default mode network (DMN) through resting‐state functional connectivity (FC) analyses (Fox & Raichle, 2007; Raichle, 2015; Raichle et al., 2001; Stawarczyk et al., 2011)—they lack sufficient specificity to capture the time‐sensitive neural dynamics of online language learning. We therefore propose a more tailored paradigm in which participants perform language learning tasks while in the fMRI machine. This would capture neural response corresponding to subjects’ linguistic performance in real time. We employed task‐based dynamic brain FC analyses, as it is the most appropriate target measurement used in studies featuring similar study designs and analytical purposes (Fong et al., 2019). On the behavioral end, by exposing participants to a continuous stream of language stimuli, we explore what synchronization across brain areas supports more or less self‐paced learning through immersion (Wong et al., 2014).

Task‐induced FC has been suggested to play an important role in conventional fMRI research (Bassett et al., 2006; Eguíluz et al., 2005; Hutchison et al., 2013; Preti et al., 2017), and a recent review by Gonzalez‐Castillo and Bandettini (2018) recapitulates the main features of this network connectivity: i) the DMN most characterizing the resting‐state connectivity can still be identified during task performance with executive control networks (ECNs) (Cole et al., 2014; Krienen et al., 2014); ii) there is an overall increase in across‐network connectivity, specifically in domain‐specific across‐network connectivity, that is positively correlated with task performance (Cole et al., 2014; Shine et al., 2016); and iii) dynamic FC patterns reveal performance quality, which allows inferences of interindividual differences in perception, learning, and other cognitive abilities (Deng et al., 2016; Gonzalez‐Castillo & Bandettini, 2018; Kepinska et al., 2017; Mennes et al., 2010). In particular, spontaneous cognitive processes that typically reflect resting scans (Hurlburt et al., 2015), such as slow rumination, self‐interrogation in interior monologue, repeated memorization, and recall, might also be observed in individuals who surrender their ears to an immersive flow of words with a certain rhythm (Simony et al., 2015); the study of spontaneous cognitive processes would be effective for elucidating the interlanguage schemas in USL and SL.

When tapping into the process of schema update, an FC analysis‐based approach would also provide insight into connectionist modeling because the artificial neural network must have approximate counterparts in the real brain systems. Several prior studies have put forth principles of correspondence, including the dual‐path model of a recurrent neural network (Chang, 2002; Chan et al., 2006). Such a model prevails due to its potential to reflect any possible viewpoints that could be postulated in linguistic processing while allowing for the identification of underlying neural mechanisms. Future studies can consider this neural network perspective with the goal of characterizing task performance and its underlying processes by mapping brain regions to nodes in an artificial neural network. However, the neural basis of schema updating, which would provide insights into the connectionist modeling, has yet to be uncovered. Looking toward the future of artificial intelligence, this study attempted to elucidate how task‐induced functional connectivity—especially DMN—could be modulated in the brain as a result of the schema update achieved by superlearning in language acquisition, which would be formed by the alternation of USL and SL and controlled by transitional probabilities between patterns for word identification.

## 2 MATERIALS AND METHODS

### 2.1 Overview

It is acknowledged that the online language learning paradigm in fMRI sessions should be designed to meet requirements of simplicity and distinctness in terms of syntactic rule and phonetic regularity. Designed for this purpose, artificial languages have often been used in neurolinguistic experiments that apply mathematical approaches to probe probabilistic sequential pattern learning (Dienes et al., 1995; Kepinska et al., 2017). In our fMRI test, in which we use an artificial grammar language as a proxy of second language (Ettlinger et al., 2016), a USL run is provided at first with a meaningless sound stream composed of sequences of syllables. We ask participants to detect patterns of syllables that they consider to be “words” without revealing the correct segmentation pattern: participants choose their preferred “word” from each of six pairs of syllable patterns in the subsequent testing block. From this first test trial, we attempt to identify a particular characteristic of statistical learning that might depend on a subjective difference in probability, or “preference” in decision‐making, in implicit but unsupervised sequential learning (Folia et al., 2011; Kaufman et al., 2010; Zizak & Reber, 2014). Here, any disyllabic word segmentation that the candidate extracts from the artificial speech follows either the high/low or low/high appearance frequency patterns, stemming from the candidate's bias toward forward and backward transitional probabilities and pitch patterns that relate to the stress position in each word candidate. This USL run is followed by three consecutive SL runs with different auditory materials, all of which conform to a structural pattern rule that remains identical throughout the whole experiment. For each SL run, prior to presenting the continuous syllable stream, six disyllabic words to be learned in that particular run are introduced as correct answers to be chosen in the ensuing test with the same alternative form as the preference selection in the USL run. The upcoming and final USL run, identical to the one presented at the very start of the experiment, completes the experiment while demonstrating how the previous three SL runs affected the participant's initial schema, effectively changing their responses without being noticed.

In this study, each subject's preference in word segmentation revealed through the first USL runs represents his/her own covert schema or interlanguage. The subsequent SL runs specify pseudowords that follow the same canonical schema (without the subject being fully aware) throughout the rest of the experiment, maintaining one of the coherent interpretations of the artificial grammar. The private schema of each subject, activated in the USL runs, might either accord with or contradict the “teacher signal word” that subjects are coerced to learn and recognize in the SL runs, although they might be unaware of it despite a gradual influence of the hidden oriented pattern control. If there is a subject‐level match between the subject's inherent USL schemas and those provided during SL, the schema is “accreted” and reinforced throughout the entire experiment, allowing the subject to learn the new language comparatively effortlessly. Otherwise, they will be obliged to thoroughly “tune” and even “reconstruct” the individual schema to be more or less in line with (or even against) the latent but objective presence of the linguistic rules presented. In short, dynamic FC enables the detection of the brain networks involved in maintaining or updating schemas between USL and SL of a language that are contingent upon characteristics specific to the individual.

### 2.2 Participants

The study participants were 22 Japanese university students (13 male, 9 female; aged 20–27 years and all right‐handed) who attended the Kenshinkai Tokyo Medical Clinic, Japan. Ethical approval was obtained from the Human Investigation Committee of Tokyo Institute of Technology. All volunteers signed a written informed consent form. Prior to the fMRI session, a General Self‐Efficacy Scale questionnaire was administered to all study participants to glean information on their characteristics. All subjects were presented with USL demonstration material, containing sound stream and disyllabic combinations that were different to those used later on in the actual run, before entering the scan room. This experience was provided so that the study participants could understand the task procedure easily but without giving any hint of the structure of the test material, except the knowledge that the word candidates were all disyllabic and embedded several times within the exposure (learning) phase. At the end of the session, the subjects were asked to complete a post hoc survey to determine how they evaluated their performance in the experiment. The survey included items that queried the quality of the participants’ performances in the first USL, the SL, and the second USL. We also asked participants to report using the 4‐point Likert scale the extent to which they were convinced that they faithfully identified the “true” words which appeared in the USL sessions. Data for two male subjects were excluded from the analysis because of excessive head motion (*n* = 1) and falling asleep during the scan (*n* = 1).

### 2.3 Task design

This study replicated the experiment on implicit statistical learning ability (Mirman et al., 2008; Perruchet & Desaulty, 2008; Xie, 2012; Yim & Windsor, 2010) performed by Onnis and Thiessen (2013) and included the same experimental task for the USL runs with the addition of new factors: namely, prosodic variables and explicit SL runs. The original unsupervised artificial grammar learning experiment aimed to detect the sensitivity of the subjects to patterns of forward‐backward transitional probabilities as a function of language background and environment through the task of finding word boundaries in a continuous speech stream designed on an AG (Onnis et al., 2015). According to Onnis et al., the forward transition probability can be defined for any given sequence of elements XY as the probability of Y given X, calculated by normalizing the co‐occurrence frequency of X and Y by the frequency of X. In contrast, the backward conditional probability for the sequence of XY is the probability of X given Y, so is calculated by normalizing the co‐occurrence frequency of X and Y by the frequency of Y. As a consequence, the combination of low frequency followed by high frequency syllables is defined as S‐HiLo, and vice versa. We slightly revised the terminology of Onnis et al., such that the “forward low‐backward high” probability was abbreviated as “syntactically HiLo” or “S‐HiLo” and “forward high‐backward low” probability as S‐LoHi. The reason for this modification was that in the present fMRI experiment, another HiLo or LoHi contrast, “prosodic‐HiLo” (“P‐HiLo”) or “prosodic‐LoHi” (“P‐LoHi”), was also implemented from the information of pitch patterns that varied the stress location in each word candidate unit. Note that because we took advantage of a text‐to‐speech engine designed to replicate the specific phonation features of each language in our study, we had no rigid or consistent distinction between “pitch,” “accent,” and “stress,” and these phonetic factors for differentiating P‐Hi and P‐Lo were configured to render some independent syllables more explicit as “Hi” than “Lo” in each language. Some syllables are more easily interpreted as P‐Hi or Pi‐Lo (they tend to fall at the extremes of the pitch spectrum and are therefore more easily classified) compared to other syllables due to their intrinsic phonetic characteristic.

We faithfully recreated the experimental design of Onnis et al. in the first and last USL runs, when subjects underwent a word segmentation activity for deciding ambiguous lexical borders within a text spoken in similar types of artificial languages. Given the equal frequency of Hi‐Lo and Lo‐Hi patterns in the unsupervised training material, there was no “correct” extraction criterion in the USL for splitting the units from the sound stream, revealing an individual preference of the underlying syllable frequency and/or stress distribution patterns. The auditory stimuli were transmitted to each participant via the MRI‐safe Serene Sound system (Resonance Technology Inc., Northridge, CA, USA). In the learning phase, subjects were prompted to detect compact sound sequences in a continuous syllable stream that were noticed multiple times, thus seeming optimal to be recognized as “word” instances. In the testing phase that followed soon after exposure to the speech stream, 12 pairs of disyllabic word candidates were provided in succession, prompting the participant to select one or the other as a valid word that they extracted from the learning phase; that is, each set of 12 forced choices was performed after the subjects had compared two disyllabic items representing “S‐HiLo” and “S‐LoHi” patterns (or vice versa, with equal probability of appearance) in turn. Subjects held a two‐button MRI‐safe fiberoptic response pad (Current Design Inc. 932‐fORP) in their left hand and were instructed to press either the yellow button with the index finger if they chose the first item or the blue button with the middle finger if they chose the second. When pairs of disyllabic units were presented for comparison, the stress was always assigned to the first syllable, so there could have be some units where the stress position was different between their presentation during the learning and testing phases.

In the USL runs, with a voice of moderate modulation, words with “forward high‐backward low” probability, that is, S‐HiLo, all have prosodic HiLo patterns (P‐Hilo). This means that their stress position in the learning phase remains the same as that in the testing phase. However, the “forward low‐backward high” probability is phonologically implemented in the converse order between the learning and testing phases so might confound the judgment of learners who inherently prefer the S‐LoHi and/or P‐LoHi pattern (since all the word candidates proposed during testing were P‐HiLo). Taking into account such inconsistency at the pronunciation level, we use the brevity code of “‐l” for listening and “‐t” for testing to express the exact stress information, such as P‐HiLo‐l, P‐LoHi‐l, and P‐HiLo‐t (with no instance of P‐LoHi‐t). The three SL runs shared the same transitional probability patterns but subjects were requested to learn six preselected disyllabic words in advance whether or not they liked them. The correct “words” to be memorized were all of the S‐HiLo pattern and, unlike the word candidates in USL, the language of which lacked rich intonation, their prosodic patterns in the speech streams were diverse, including prosodically neutral ones, so the prosodic information itself did not provide particularly decisive cues for detecting word boundaries. The task of subjects in the SL runs was to recognize the learnt words in the listening part and perform the recall test, the form of which was identical to that of the USL runs.

**FIGURE 1:**
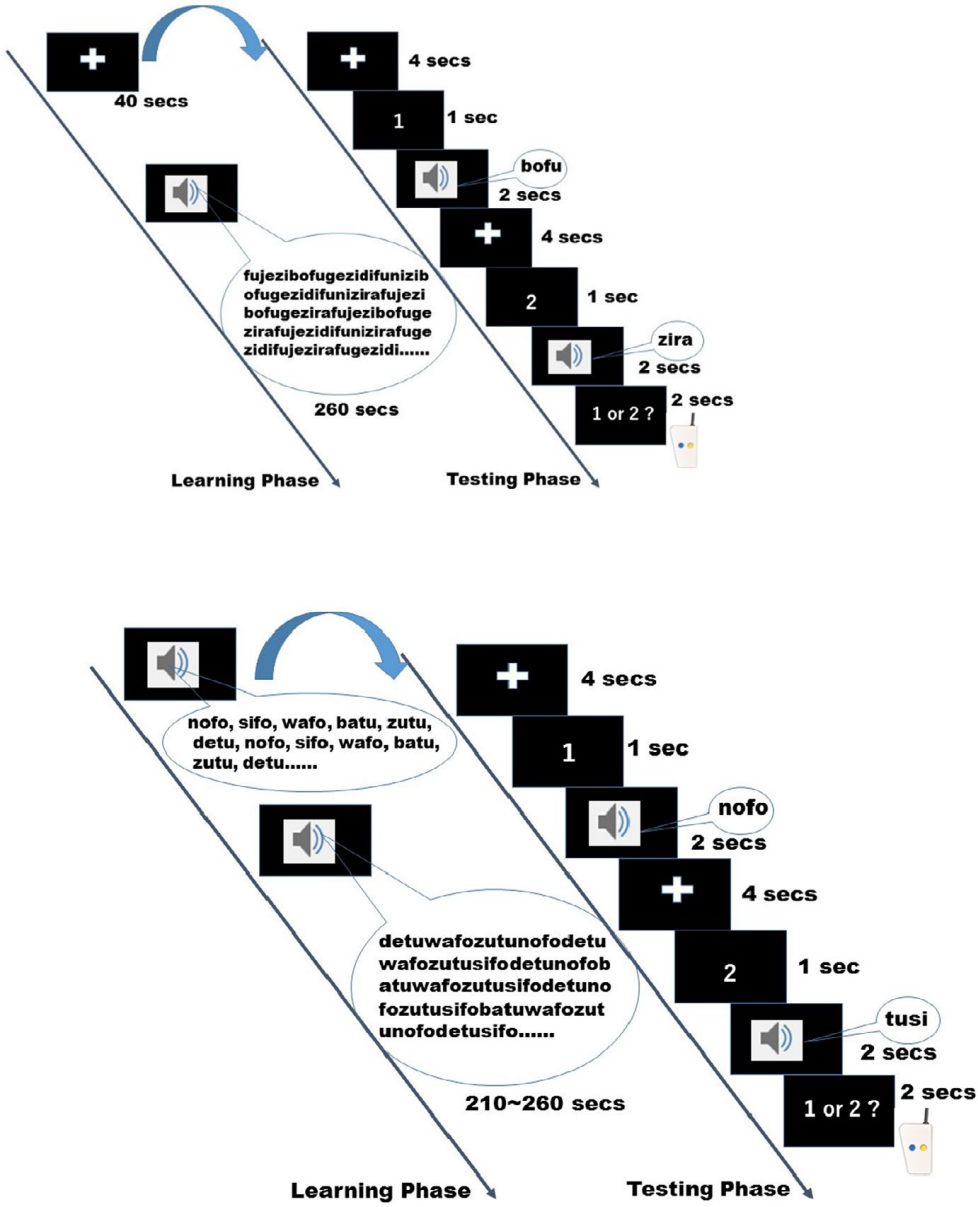
Time courses of the USL and SL runs. Above: Time course of the USL runs voiced by Veena. Below: Time course of the SL runs with the voices of Amelie (featured in this figure), Anna, and Luciana, for each of the three runs, respectively. They are composed of a learning phase that differs by the opening stimulus (a 40‐s resting state for USL and repeated presentation of correct words—all “S‐HiLo”—for SL), and a following testing phase. SL, supervised learning; USL, unsupervised learning

In summary, each fMRI session was composed of five runs in the order of one USL run, three SL runs, and a repetition of the first USL run. In the SL runs, different stimulus sets were provided in a fixed order across participants. Each run was subdivided into a learning (listening) phase and a testing phase. In the USL runs only, the learning phase was preceded by a resting‐state scan for 40 s with a fixation cross mark at the center of the screen. This part of the USL was replaced with instruction of correct words in SL, in which six S‐HiLo words were sequentially presented six times. The forced choice task was carried out at the end of each run to evaluate the articulation preference in the USL and the memorization accuracy in the SL. The auditory and orthographic stimulus creation, playback, and behavioral response recordings were made using E‐Prime 2.0‐Standard (Psychology Software Tools, Inc., Sharpsburg, PA, USA). The time course of a trial for the USL and SL is depicted in Figure 1.

### 2.4 Stimulus set

#### 2.4.1 Learning phase

Although these frequency patterns were fixed in this experiment, the particular set of stimuli involved were entirely different across runs except for the two USL sessions. The integer at the end of each pattern string is the number of a word candidate in an artificial‐language sample set. Each sound stream in the learning phase was created by the “say” command of MAC OS using different synthesized female voices (“Veena” as a voice for Indian English in USL; “Amelie,” “Anna,” and “Lucianna,” respectively representing Canadian French, German, and Brazilian Portuguese in SL) with the speech rate set to 200. It consisted of the following continuous sequence based on the rules of a stochastic Markovian grammar chain conceived by Onnis et al.: X, a1, Y, b1, X, a2, Y, b3, X, a3, Y, b1, X, a2, Y, b3, X, a3, Y, b2, X, a1, Y, b1, X, a2, Y, b2, X, a1, Y, b1, X, a2, Y, b2, X, a1, Y, b3, X, a3, Y, b2, X, a2, Y, b3, X, a1…

The probabilities of appearance of X and Y (highly frequent) were both 25% through one learning sound stream and the remaining half of the syllables were almost equally assigned to a1, a2, a3, b1, b2, and b3 (infrequent). This sequence can be segmented into either of the following syllable patterns. Note that ‘Hi’/’Lo’ does not mean a high/low frequency of appearance; on the contrary, ‘S‐HiLo’ as a whole represents the “forward high‐backward low” probability whereas ‘S‐LoHi’ represents the “forward low‐backward high” probability in parsing.

S‐HiLo.1: ‘b1 X’; S‐HiLo.2: ‘b2 X’; S‐HiLo.3: ‘b3 X’; S‐HiLo.4: ‘a1 Y’; S‐HiLo.5: ‘a2 Y’; S‐HiLo.6: ‘a3 Y’.

S‐LoHi.1: ‘X a1’; S‐LoHi.2: ‘X a2’; S‐LoHi.3: ‘X a3’; S‐LoHi.4: ‘Y b1’; S‐LoHi.5: ‘Y b2’; S‐LoHi.6: ‘Y b3’.

The stimulus materials are summarized in Table 1. The concrete stimulus materials were as follows.

**TABLE 1:** Stimulus Materials Provided to Participants During Artificial‐Grammar Learning and Testing Phases

	USL run	SL run1	SL run2	SL run3	USL run
a1	je	ba	ve	chi	je
a2	ge	zu	ti	se	ge
a3	ni	de	lu	mo	ni
b1	bo	no	ro	pu	bo
b2	ra	si	so	na	ra
b3	di	wa	ka	li	di
X	fu	fo	pi	bo	fu
Y	zi	tu	she	wi	zi

voice	Veena	Amelie	Anna	Luciana	Veena
rest	40,000	0	0	0:00	40,000
total time train(ms)	260,000	260,000	210,000	235,000	260,000
total vols(TR=1)	300	260	210	235	300
total scan time	5:00	4:20	3:30	3:55	5:00
fw					
S‐HiLo 0.1 b1 X	**bo**fu	**no**fo	ro**pi**	**pu**bo	**bo**fu
S‐HiLo 0.2 b2 X	**ra**fu	sifo	so**pi**	**na**bo	**ra**fu
S‐HiLo 0.3 b3 X	**di**fu	**wa**fo	**ka**pi	**li**bo	**di**fu
S‐HiLo 0.4 a1 Y	**je**zi	batu	veshe	chiwi	**je**zi
S‐HiLo 0.5 a2 Y	**ge**zi	zutu	tishe	sewi	**ge**zi
S‐HiLo 0.6 a3 Y	**ni**zi	detu	lushe	mowi	**ni**zi
bk					
S‐LoHi 0.1 X a1	fu**je**	foba	**pi**ve	bochi	fu**je**
S‐LoHi 0.2 X a2	fu**ge**	fozu	**pi**ti	bose	fu**ge**
S‐LoHi 0.3 X a3	fu**ni**	fode	**pi**lu	bomo	fu**ni**
S‐LoHi 0.4 Y b1	zi**bo**	tu**no**	shero	wi**pu**	zi**bo**
S‐LoHi 0.5 Y b2	zi**ra**	tusi	sheso	**wi**na	zi**ra**
S‐LoHi 0.6 Y b3	zi**di**	tu**wa**	she**ka**	**wi**li	zi**di**

Note

A bold font is used for listing the materials in the testing phases and to indicate the locations of prosodic stress within each of the disyllabic items within the speech stream of the learning phases (during testing, the stress was consistently given to the first syllable).

Abbreviations: SL, supervised learning; USL, unsupervised learning.

The first and last USL run:

fujezibofugezidifunizibofugezidifunizirafujezibofugezirafujezibofugezirafujezidifunizirafugezidifujezirafugezidi…(Voice: Veena, duration 260 s)

First SL run:

detuwafozutunofodetuwafozutusifodetunofobatuwafozutusifodetunofozutusifobatuwafozutunofodetusifozutu…(Voice: Amelie, duration 260 s)

Second SL run:

lushekapitisheropilushekapitishesopilusheropiveshekapitishesopilusheropitishesopiveshekapitisheropilushesopitishe…(Voice: Anna, duration 210 s)

Third SL run:

bochiwipubosewimabomowipubosewimabomowinabochiwipubosewinabochiwipubosewinabochiwimabomowinabosewimabochi…(Voice: Luciana, duration 235 s)

The gothic fonts used in the list of word candidates in Table 1 imply the position of stress in the continuous speech stream, so that the word corresponding to S‐HiLo.1 ‘b1 X’ in the SL run 2, that is “ropi,” for example, has a prosodic emphasis at the second syllable “pi” during the listening phase as an instance of “P‐LoHi‐l.” Thus, this word was heard by the subjects with different pronunciations between the learning and testing phases because any disyllabic word when cut up in the testing had an initial stress as “P‐HiLo‐t.” The words without gothic fonts were more or less phonetically monotonous in the learning. Noteworthy is that the Indian English voice “Veena” used for the USL sessions solely presented a consistent prosodic stressing rule, enabling us to adjust the syntactic and prosodic patterns such that all the S‐HiLo words were P‐HiLo and all the S‐LoHi words were P‐LoHi in our learning phase dataset.

The number of syllables was always 524 across the training sets but those contained within approximately 8 s at the beginning and ending of each sequence were more or less difficult to hear under the fade‐in and fade‐out effect used to eliminate hints (temporal bias) for detecting word boundaries. Although the speech rate was always set to 200, the total time lengths were different because of the specifics of the voices generated by the “say” program (minimum 210 s, maximum 300 s). Therefore, the numbers of scan volumes differed across runs. Our artificial grammar samples shared no surface features and were meticulously constructed to eliminate cues for phonetic or conceptual association due to a resemblance to real Japanese words.

#### 2.4.2 Testing phase

There were 12 testing trials posterior to the learning phase of each run. We induced in this phase a set of forced choices to be performed after letting the subjects compare two disyllable items representing “S‐HiLo” and “S‐LoHi” patterns (or vice versa, with equal probability of either order being presented) in turn. Each subject held an MRI‐safe response pad in the left hand and was requested to press either the yellow button by the index finger if choosing the first item or the blue button by the middle finger if choosing the second one. The time course for each trial was as follows. The subject was asked to perform the forced choice after being presented with the second disyllabic word (which had a 50% chance being S‐HiLo or S‐LoHi), and the subject was not allowed to make choices before presentation of both words was completed (Table 2).

**TABLE 2:** Time course of the testing phase

+(Fixation)	4 s
Prompt (1)	1 s
Sound (1)	2 s
+ (Fixation)	4 s
Prompt (2)	1 s
Sound (2)	2 s
Questions [(1) or (2)?]	2 s
Total	16 s

All the testing sets across the five runs consisted of the following 12 ordered combinations but using completely different voice materials. The integer at the end of each pattern string represents each word candidate number in an artificial language sample set. Table 3 represents the order of the samples provided in each testing trial.

**TABLE 3:** Twelve patterns of forced choice task administered during the testing phase of each run and their order of presentation

Test 1: S‐HiLo5, S‐LoHi5
Test 2: S‐HiLo1, S‐LoHi1
Test 3: S‐LoHi2, S‐HiLo3
Test 4: S‐HiLo2, S‐LoHi2
Test 5: S‐HiLo4, S‐LoHi4
Test 6: S‐LoHi3, S‐HiLo4
Test 7: S‐HiLo6, S‐LoHi6
Test 8: S‐LoHi5, S‐HiLo6
Test 9: S‐LoHi1, S‐HiLo2
Test 10: S‐LoHi6, S‐HiLo1
Test 11: S‐LoHi4, S‐HiLo5
Test 12: S‐HiLo3, S‐LoHi3

### 2.5 Data acquisition

Functional MRI scans were acquired with a 3.0‐T scanner Magnetom Skyra (Siemens, Erlangen, Germany) and 32‐channel head coil at the Kenshinkai Tokyo Medical Clinic, Japan. Functional scanning was performed using an EPI sequence (“ep2dbold”) with a 1000‐ms repetition time, a 22‐ms echo time, and a 62‐degree flip angle. Using a Multi‐Band Accelerated EPI Pulse Sequence, developed by the Center for Magnetic Resonance Research at the University of Minnesota, with the multiband factor set to 6, each volume consisted of 66 slices, each 2‐mm thick, with a distance factor of 15 percent and a voxel size of 3.0 × 3.0 × 2.0 mm. We readapted our 3.0‐T multiband MRI scanner to the optimized EPI protocol developed by the research group at the Center for Mind/Brain Sciences, University of Trento, for decreasing the susceptibility loss effects in the anterior temporal lobe and increasing the time‐series signal‐to‐noise ratio of the BOLD effect for their 4.0‐T Bruker scanner with an 8‐channel coil (Gesierich et al., 2012). It has been acknowledged that the anterior temporal lobe is a hub locus responsible for combining information in semantic processing, and this functional role is played by a direct nerve bundle connection to the left inferior frontal gyrus, that is, the conventional Broca's area involved in language. We could replicate, with the shortest echo time (22‐ms) comparable to that used in the Center for Mind/Brain Sciences protocol, the same inline resolution and a slice thickness (2.0 mm) that was smaller than the pixel size in the phase and frequency encoding directions (3.0 mm). As in the study by Gesierich et al., (2012), axial slices were oriented approximately –20 degrees relative to the anterior commissure‐posterior commissure plane, thereby approximately parallel to the longitudinal axis of the temporal lobes. The multiband factor 6 enabled full‐brain coverage with an interleaved slice acquisition timing order of [0, 532.5, 87.5, 622.5, 177.5, 712.5, 267.5, 800, 355, 890, 445] as a millisecond unit of time vector repeated six times to obtain 66 slices within a whole volume. Note that we adopted AP (anterior to posterior) as the direction of phase encoding after examining the quality of field maps for AP and PA (posterior to anterior). The parameter values set for the anatomical scans (using “magnetization prepared rapid gradient echo [MP‐RAGE]”) included a repetition time of 1,800 ms, an echo time of 2.50 ms, a flip angle of 9 degrees, a bandwidth of 250 Hz/Px, and an isotropic voxel size of 0.8 mm.

### 2.6 Methodology

The initial data processing was performed through the default preprocessing pipeline for volume‐based analyses (direct normalization to the MNI space) of the CONN‐fMRI functional connectivity toolbox v18a (www.nitrc.org/projects/conn, RRID: SCR_009550) using Statistical Parametric Mapping software (SPM12; Wellcome Department of Cognitive Neurology; London, UK) with its default tissue probability maps. Functional data were motion‐corrected by realignment and unwarp functions and were centered to the coordinates of the original point [0 0 0] before moving on to slice‐timing correction based on the aforementioned slice acquisition timing order. Artifact Detection Tools (ART)‐based identification of outlier scans was used for scrubbing. Simultaneous segmentation of gray matter, white matter, and cerebrospinal fluid and standard Montreal Neurological Institute (MNI) normalization with a resliced isotropic voxel size of 2 mm were performed subsequently. Structural data were translated to the coordinates of the original point [0 0 0] and became the target of segmentation and normalization with a resliced isotropic voxel size of 1 mm. For the functional outlier search, we used intermediate settings of 97th percentiles in a normative sample, and functional smoothing via spatial convolution was performed with an 8‐mm Gaussian kernel.

FC analyses were also carried out for the learning phase (excluding the initial resting states of the USL runs, the teacher signal presentation of the SL runs, and the testing phase) using the CONN toolbox v18a, with the default frequency band of the CONN (<0.10 Hz). The connectivity strength between the source and target ROIs was statistically evaluated based on the CONN brain atlas, named “atlas.nii” and “networks.nii” from the FSL Harvard‐Oxford Atlas, as well as the cerebellar areas from the AAL Atlas (Whitfield‐Gabrieli & Nieto‐Castanon, 2012). The group‐level connectivity matrix for 132 ROIs and 32 representative nodes of the eight intrinsic networks (default mode, sensorimotor, visual, saliency, dorsal attention, frontoparietal, language, and cerebellar) was computed for assessment of the main effects of USL‐SL‐USL artificial language training and their differences.

We mainly used the ROI‐to‐ROI connectivity analysis to evaluate the magnitude of t‐contrasts between learning conditions. Given that the aim of our research is to explore the neural basis of broad general concepts (“meta‐concepts”) that can be recapitulated by the abstract term “schema,” it is neither worthwhile to pre‐determine a few seeds to serve as important nodes nor to extract significant voxels within each of the target regions scrutinizing tables of functional anatomy. Hence, we analyzed the FC graphs of nodes in the whole region and performed an exhaustive assessment of the overall edges using *t* tests corrected for multiple comparisons. The significant ROI‐to‐ROI connectivity was thus taken as the neural “representation” of each learning method with its corresponding schema; this representational difference between USL and SL is viewed as the modulation of schema brought about by the switch of learning methodologies.

## 3 RESULTS

Upon completion of the session, no participant stated that he or she was aware of the equal frequency distributions of disyllabic combinations, that is, the objectively equal probabilities of existence of all the candidate words. Interestingly, subjects who rated their performance as poor in the first USL run tended to report being satisfied by their competence in the second (last) USL run (*r* = .739, *p* = .0002 for the correlation between the binary answers to the two questions posed about the two USL runs in reverse for the coding of yes: 1, no: 0). Nevertheless, there was no correlation between these self‐assessments of each separate USL performance and test scores in the SL run (*r* = .1 for the first USL and *r* = .204 for the last USL). Hence the accuracy of memory testing in the SL could be treated as a factor independent of self‐assessment on the preceding and following USL performances. However, there was a significant correlation between the SL test accuracy rate and the four‐scale conviction degree ratings of the subjects when asked whether or not they could finally ascertain the “assumed intended” words as a whole (*r* = .543, *p* < .05).

### 3.1 Contrast analysis between the first USL, SL, and second USL runs

By forcing the subjects to alter their learning strategies (USL versus SL), which started with USL and then moved on to three SL trials and then looped back to USL for a second time, we could set three contrasts between any two of the three merged sessions (first USL with one run, SL with three runs, and the final USL that was identical to the first one) for evaluation of the difference in strength of association across the exhaustively enumerated edges, that is, ROI pairs, via a *t* test with a corrected false discovery rate of *p* < .01: A) first USL versus last USL; B) first USL versus SL, and C) last USL versus SL. The results of the ROI‐to‐ROI analysis for these contrasts are as follows and listed in Table 4.

- The between‐region links that were significantly stronger for the first USL than for the SL were between i) the right inferior frontal lobe, pars opercularis, and visual‐occipital networks [0,93,−4], (*t* = 5.38 p‐FDR = 0.006), ii) the left inferior frontal lobe, pars opercularis, and left amygdala (*t* = 5.8, p‐FDR = 0.002), and iii) the left inferior frontal lobe, pars opercularis, and left posterior middle temporal gyrus (*t* = 5.28, p‐FDR = 0.007), in the order of seed/sources and targets, and vice versa. No association was significantly larger for the SL than for the first USL (*t* < 0). Note that the contents of the two USL runs were exactly the same and only the order effect enhanced by the SL exercises gave rise to the contrasts.
- The ROI‐to‐ROI contrasts between the first USL and the following SL were mainly characterized by involvement of the DMN nodes, which were more synchronously activated in the first USL. The precuneus, which is the most crucial region for the DMN system (Utevsky et al., 2014) was featured by its links with the left lateral parietal portion in the DMN [−39, −77, 33] (*t* = 8.83, p‐FDR = 0.000015), the right lateral parietal portion in the DMN [47, 67, 29] (*t* = 5.25, p‐FDR = 0.003888), and the posterior cingulate cortex portion in the DMN [1,‐61, 38] (*t* = 4.77, p‐FDR = 0.00755). The ROI‐to‐ROI contrasts with significant negative t‐values could be detected between each of the bilateral juxtapositional lobules (formerly known as the supplementary motor cortex [SMA]) and the posterior portion of the left superior temporal gyrus (*t* = −5.87, p‐FDR = 0.000674 for SMA *r* and *t *= −4.77, p‐FDR = 0.00474 for SMA l).
- We extracted a variety of connections belonging to several functional (sub)networks to compare the second USL run with the preceding SL runs. An auditory occipital association (Cate et al., 2009) was detected between Heschl's gyri (auditory cortex) and the lingual gyri (visual cortex) in the second USL run whereas the associations in the SL runs were found to be between the right lingual gyrus and right Heschl's gyrus (*t* = 5.28, p‐FDR = 0.003624) and between the right lingual gyrus and right Heschl's gyrus (*t* = 5.3, p‐FDR = 0.002361). Moreover, in addition to the modality‐specific connectivity, language‐related, salience, or miscellaneous networks were detected in the third contrast of the second USL >SL, that is, the bilateral superior parietal lobules, which were connected to the right inferior frontal gyrus (homologous portion of Broca's area) or the left anterior insula representing the salience network (SN). For example, the inferior frontal gyrus and right pars opercularis as the seed showed significant connections with the ROIs in the right superior parietal lobule (*t* = 5.28, p‐FDR = 0.007237) and the left superior parietal lobule (*t* = 4.87, p‐FDR = 0.008889). The right superior parietal lobule assumed in turn the role of seed for a significant associativity with the left anterior insula as a node of the SN at [−44, 13, 1] (*t* = 5.43, p‐FDR = 0.005208). The right juxtapositional lobule was connected with the superior sensorimotor network at [0, −31, 67] (*t* = 5.35, p‐FDR = 0.006151).

**TABLE 4:** Results of ROI‐to‐ROI connectivity analysis for contrast between first unsupervised learning, the supervised learning, and second unsupervised learning

1st USL > 2nd USL			
Seed/Sources	Targets	T	p‐FDR
atlas.IFG operr	networks.Visual.Occipital(0, 93,‐4)	5.38	0.00585
atlas.IFG operl	atlas.Amygdala l	5.8	0.002324
	atlas pMTGl	5.28	0.007244
atlas pMTGl	atlas.IFG operl	5.28	0.007244
atlas.Amygdala l	atlas.IFG operl	5.8	0.002324
networks.Visual.Occipital(0,93,‐4)	atlas.IFG operr	5.38	0.00585

1st USL > SL			
Seed/Sources	Targets	T	p‐FDR
atlas.pSTG l	atlas.SMAr(Juxtapositional Lobule Right)	‐5.87	0.000674
	atlas.SMAl(Juxtapositional Lobule Left)	‐4.77	0.00474
	networks.FrontoParietal.LPFC (R)(41,38,30)	4.69	0.004516
*atlas.SMAr*	atlas.pSTGl	‐5.87	0.002022
atlas.AC(Cingulate Gyrus)	atlas.Por(ParietalOperculum)	5.15	0.00961
atlas.Precuneous	networks.DefaultModeLP(L)(‐39,‐77,33)	8.83	0.000015
	networks.DefaultModeLP(R)(47,67,29)	5.25	0.003888
	networks.DefaultModePCC(1,‐61,38)	4.77	0.00755
atlas.Por(ParietalOperculum)	atlas.AC(Cingulate Gyrus)	5.15	0.00961
atlas.Thalamusr	atlas.pTFusCr	5.83	0.002174
networks.DefaultModeLP(L)(‐39,‐77,33)	atlas.Precuneous	8.83	0.000011
	networks.DefaultModePCC(1,‐61,38)	8.12	0.000011
networks.DefaultModeLP(R)(47,67,29)	atlas.Precuneous	5.25	0.004411
	networks.DefaultModePCC(1,‐61,38)	5.19	0.004411
networks.DefaultModePCC(1,‐61,38)	networks.DefaultModeLP(L)(‐39,‐77,33)	8.12	0.000023
	networks.DefaultModeLP(R)(47,67,29)	5.19	0.004411
	atlas.Precuneous	4.77	0.00755
	atlas.Por(ParietalOperculum)	4.38	0.009397

2nd USL > SL			
Seed/Sources	Targets	T	p‐FDR
atlas.IFGoperr	atlas.SPLr(Superior Parietal Lobule Right)	5.28	0.007237
	atlas.SPLi(Superior Parietal Lobule Left)	4.87	0.008889
atlas.SPLr(Superior Parietal Lobule Right)	network.SalienceAInsula(L)(‐44,13,1)	5.43	0.003619
	atlas.IFGoperr	5.28	0.003619
atlas.ICCr(Intracalcarine Cortex Right)	atlas.Hgr(Heschl's Gyrus Right)	5.5	0.002245
atlasSMAr(Juxtapositional)	networkSensoriMotor.superior(0,‐31,67)	5.35	0.006151
atlas.LGr(LingualGyrusRight)	atlas.HGr(Heschel's Gyrus Right)	5.28	0.003624
atlas.LGl(LingualGyrusLeft)	atlas.HGr(Heschl's Gyrus Right)	5.3	0.002361
	atlas.HGl(Heschl's Gyrus Left)	5.29	0.002361
atlas.OFusGl(Occipital Fusiform Gyrus left)	atlas.PTl(Planum Temporal Left)	4.99	0.00683
atlas.HGr(Heschl's Gyrus Right)	atlas.ICCr(Intracalcarine Cortex Right)	5.5	0.001449
	networks.Visual.Medial(2,‐79,12)	5.35	0.001449
	atlas.LGl(LingualGyrusLeft)	5.3	0.001449
	atlas.LGr(LingualGyrusRight)	5.28	0.001449
atlas.HGl(Heschl's Gyrus Left)	atlas.LGl(LingualGyrusLeft)	5.29	0.001771
networkSensoriMotor.superior(0,‐31,67)	atlasSMAr(Juxtapositional)	5.35	0.006151
networks.Visual.Medial(2,‐79,12)	atlas.HGr(Heschl's Gyrus Right)	5.35	0.000094
network.SalienceAInsula(L)(‐44,13,1)	atlas.SPLr(Superior Parietal Lobule Right)	5.43	0.005208

### 3.2 High‐score group versus low‐score group

Next, we highlight the key findings related to how the subjects answered correctly or not before finishing each SL session. Considering the SL accuracy distribution (mean 0.72, standard deviation 0.21), we subdivided the cohort into a high‐score group of 10 subjects, certified as skilled learners, who reported with more than 80% precision, and a low‐score group of 10 subjects with less than 80% accuracy.

**FIGURE 2:**
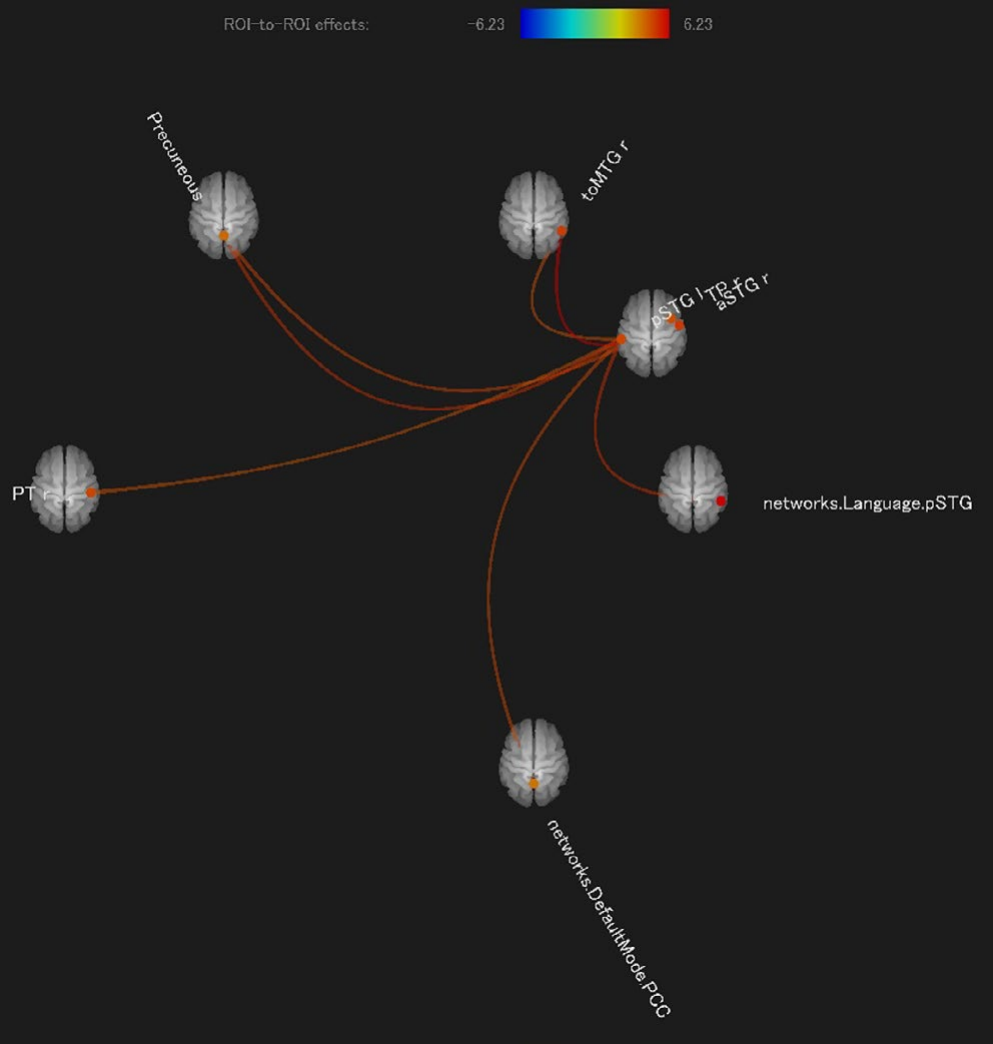
Results of connectivity analysis of functional MRI data recorded in the listening phase of the final (second) USL run between the high‐ and low‐score groups (high >low): difference in performance for tests at the end of each SL run. Graph of ROI‐to‐ROI effects (*p* <.01 FDR, two‐sided)

Reviewing the 4‐point Likert scale scores for self‐assessment of task performance in the scanner, there was a significant correlation between the accuracy scores for the post‐SL tests and the extent to which the subjects thought that they could understand the intended words as a whole, including on the USL runs (*r* = .543, *p* < .05). The apparent assumption here is that there was relationship to be explored in each subject between the discovery of knowledge during the USL run and word acquisition in the SL, despite the language of each run being independent and unlike material‐wise, except at the level of syntactic or phonetic grammar. However, it is noteworthy that, at the end of each USL, half of the high‐score group showed a tendency to choose lexical candidates with the S‐HiLo pattern, specifically used to provide the words to be learnt in the SL runs, whereas the low‐score group showed no such tendency. The precision rate in the post‐SL tests was significantly correlated with the number of times they chose the s‐HiLo pattern syllables in the first post‐USL (*r* = .498, *p* = .026) and the total two post‐USL sessions (*r* = .504, *p* = .024), although this relationship did not reach significance in the second post‐USL session (*r* = .428, *p* = .060). Table 5 enumerates the significant links (*p* <.01 FDR, two‐sided) in the ROI‐to‐ROI analysis and contrasts the performances of the high‐score group and the low‐score group from the connectivity data that targeted the listening phase of the second and final USL. We recognized that the bilateral temporal language networks from Wernicke's area were joined with the medial posterior portion of the DMN (posterior cingulate cortex and precuneus) in the brains of subjects who achieved well in SL (Figure 2). The targets for the posterior portion of the left superior temporal gyrus (i.e., Wernicke's area) as the seed were the precuneus (*t* = 5.33, p‐FDR = 0.0038), the language network in the posterior portion of the right superior temporal gyrus at [59, −42, 13] (*t* = 5.32, p‐FDR = 0.0038), the posterior cingulate cortex in the DMN at [1,‐61, 38] (*t* = 4.87, p‐FDR = 0.0069), the right temporo‐occipital portion of the middle temporal gyrus (*t* = 4.7, p‐FDR = 0.0072), and the right planum temporale (*t* = 4.59, p‐FDR = 0.0074).

**TABLE 5:** Results of ROI‐to‐ROI connectivity analysis for contrast measured in the second unsupervised learning runs between the high‐score and the low‐score groups

High score group versus low score group (in 2nd USL)			
Seed/Sources	Targets	T	p‐FDR
atlas. Precuneous	atlas.pSTG l	5.53	0.0074
	atlas.TP r	4.91	0.0092
atlas.toMTG r	atlas.aSTG r	6.23	0.0011
networks. Language.pSTG (R) (59, −42, 13)	atlas.pSTG l	5.32	0.0076
atlas.pSTG l	atlas. Precuneous	5.33	0.0038
	networks. Language.pSTG (R) (59, −42, 13)	5.32	0.0038
	netwworks. DefaultMode.PCC (1, −61, 38)	4.87	0.0069
	atlas.toMTG r	4.7	0.0072
	atlas.PT r	4.59	0.0074
atlas.aSTG r	atlas.toMTG r	6.23	0.0011

### 3.3 Schema‐matching group versus schema‐mismatching group

**FIGURE 3:**
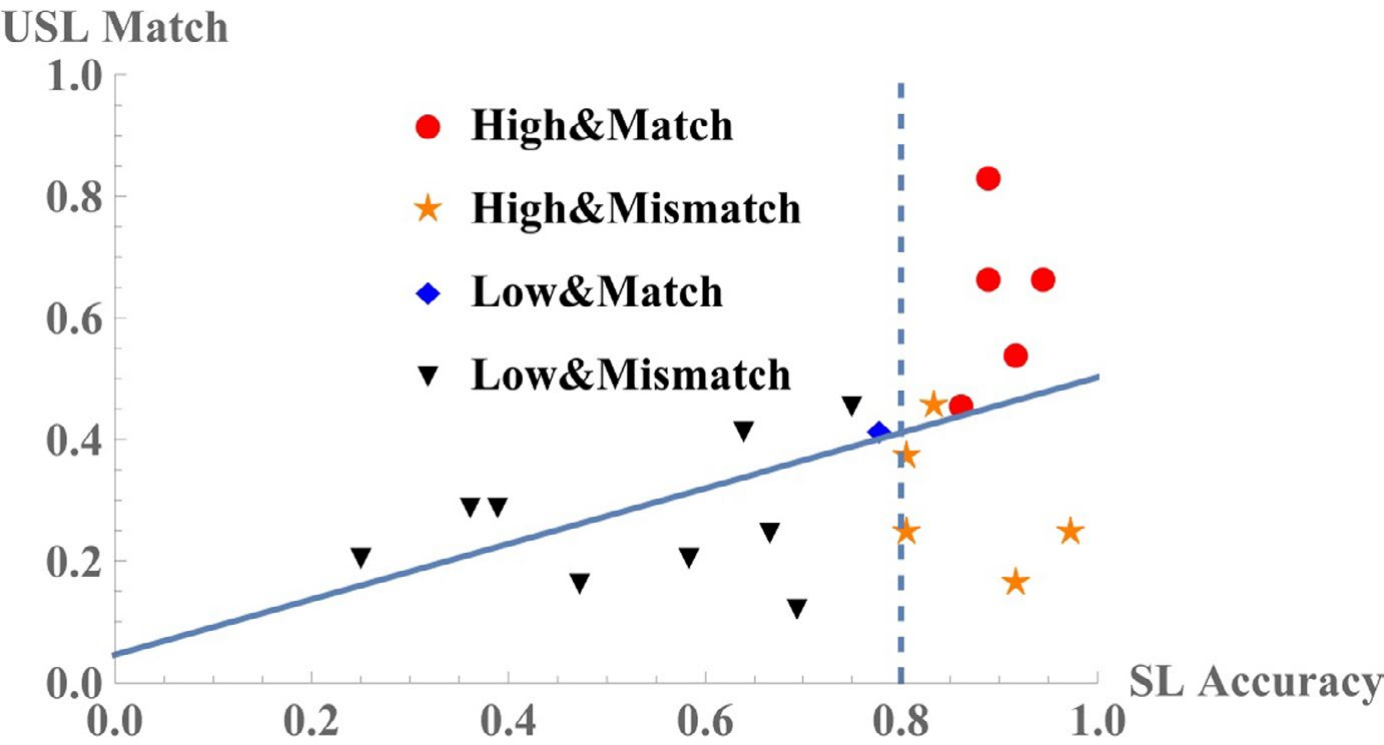
Performances of all the subjects in the USL and SL sessions. This scatter plot elicited a significant correlation between the test accuracy rates from the SL sessions (x‐axis) and the preference rates for S‐HiLo (schema matching) over S‐LoHi (schema mismatching) from the USL sessions (y‐axis). The linear regression model was: y = 0.456 x + 0.046 with a p value of 0.024 for the weight to independent variable. The Pearson's correlation coefficient between the variables (r) was 0.504 (*p* <.05). Each dot represents an individual subject classified by color and shape according to two types of grouping. The boundary between the high‐score group (abbreviated as “High”) and the low‐score group (“Low”) was set to x = 0.8 for evaluating the accuracy in the SL testing. The criterion for distinguishing the schema‐matching group (abbreviated as “Match”) and the schema mismatching group (“Mismatch”) is that the subjects belong to the former preferred and chose, at least once during the two USL runs, S‐HiLo patterns more than six times out of twelve comparison tests showing the S‐HiLo propensity, whereas those within the latter group were the remaining subjects

As previously mentioned, the preference in syntactic or prosodic patterns, which was revealed through the USL runs, is crucial for lexical acquisition in SL, where these patterns serve as hidden cues for memory retrieval. The disyllable segments with the S‐HiLo pattern shared the same accent position between learning and testing (preference disclosure) in the USL runs, but for the S‐LoHi words, the prosodic pattern was inverted to P‐LoHi for listening and P‐HiLo for testing. Therefore, the subjects were subdivided into a schema‐matching group and schema‐mismatching group according to the outcome of preference. The schema‐matching group included 6 subjects who elicited S‐HiLo propensity at least once during the two USL runs, while the schema‐mismatching group contained the remaining 14 subjects, who had never shown such behavior despite the prosodic incongruity in the opposite S‐LoHi words. All the members of the schema‐matching group belonged to the high‐score group except for one subject who had an accuracy score of 77.8%. The mean difference in SL scores between the schema‐matching group (mean accuracy 0.88, standard deviation 0.057) and schema‐mismatching group (mean accuracy 0.65, standard deviation 0.218) was significant according to the *t* test (*t* = 3.615, *p* = .002). Note that there was a significant correlation across all subjects between the accuracy rate recorded in the SL runs and the preference for S‐HiLo over S‐LoHi during the two USL runs (*r* = .503, *p* < .05). Concerning the relationship between the SL test scores and USL schema matching, the performances of all the subjects are shown in Figure 3, where each dot represents a single subject. We crossed the two binary criteria (High‐score group versus low‐score group and Schema‐matching group versus schema‐mismatching group) to classify the subjects into four subgroups. The color and shape of the dot indicate the group to which each individual belonged. This scatter plot reveals a significant correlation between the test accuracy rates in the SL sessions (x‐axis) and preference rates of S‐HiLo (schema matching) over S‐LoHi (schema mismatching) in the USL sessions (y‐axis).

**FIGURE 4:**
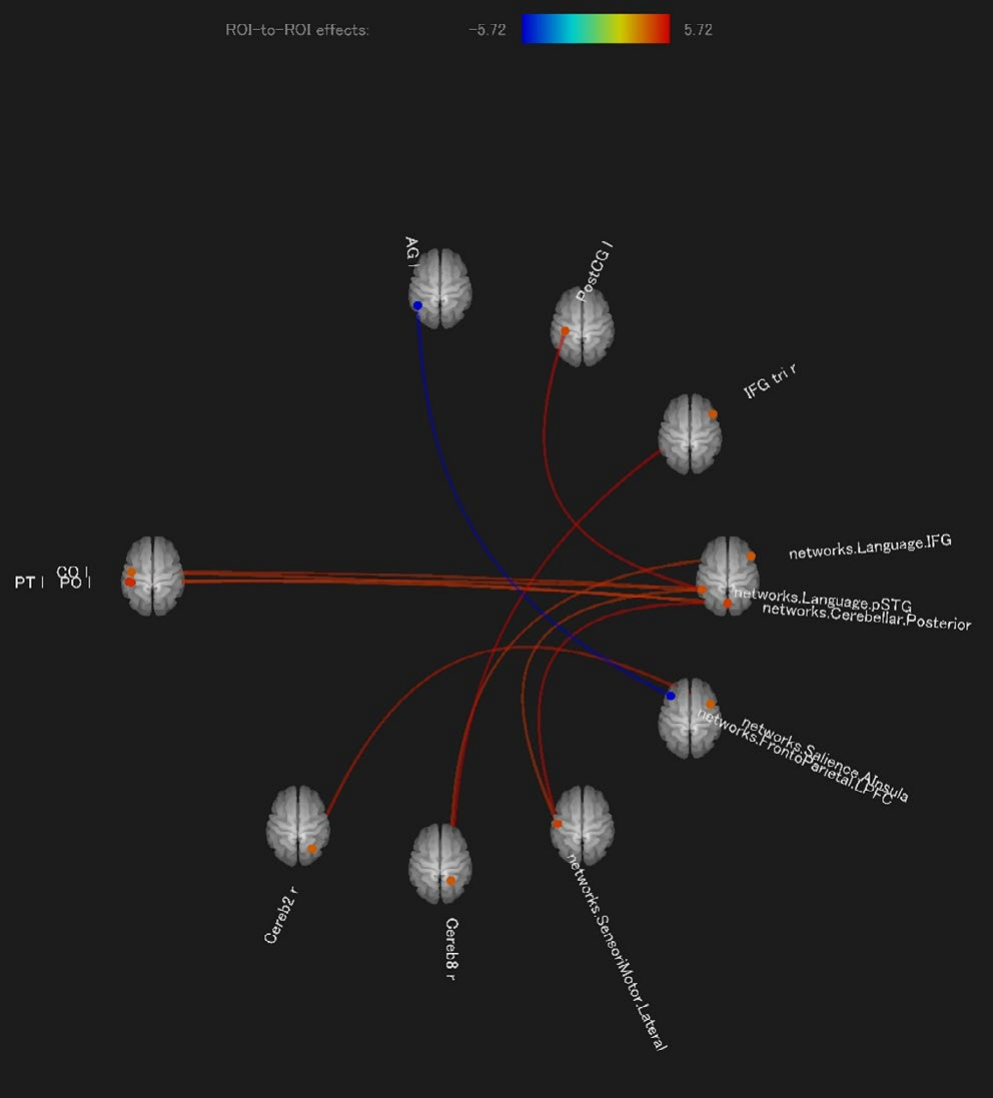
Results of connectivity analysis of functional MRI data recorded in the listening phase of the first USL run between the schema‐matching group and the schema‐mismatching group (match >mismatch): difference in choice of S‐HiLo pattern words for tests at the end of the two USL runs. Graph of ROI‐to‐ROI effects (*p* <.01 FDR, two‐sided)

Table 6 lists the significant links (*p* <.01 FDR, two‐sided) in the ROI‐to‐ROI analysis for the first USL in order to compare the network responses of the schema‐matching group and the schema‐mismatching group. The negative t‐values indicate a stronger association for the schema‐mismatching group (between the left angular gyrus and the left prefrontal cortex as a node of the frontoparietal network, presumably reflecting the anticorrelation between the task‐negative and task‐positive networks). As in the case of the group‐level test “score” contrast at the second USL, the posterior portion of the superior temporal gyrus (i.e., Wernicke's area) was identified again as a hub of the ROI‐to‐ROI‐based significant subnetwork corresponding to the group‐level “preference” contrast at the first USL, although the seed extracted here was the language‐related network areas of the posterior portion of the left superior temporal gyrus with the center at [−57, 47, 15]. However, the composition of this subnetwork (Figure 4) indicates that some parts of the cerebellum responsible for cognitive functions subserve the process of eliciting preference for schema‐matching patterns in the USL of artificial grammar languages. The posterior part of the cerebellar network at [0, −79, −32] as the seed shaped significant connections with the left lateral part of the sensorimotor networks at [−55, −12, 29] (*t* = 5.58, p‐FDR=0.0044), the left central opercular cortex (*t* = 4.88, p‐FDR=0.0089), and the left planum temporale (*t* = 4.74, p‐FDR=0.0089). Moreover, the right cerebellum 2 had a connection with the right anterior insula in the SN represented by the MNI coordinate of [47, 14, 0] (*t* = 5.27, p‐FDR=0.0084), while the right cerebellum 8 was strongly associated with the right homologous portion of Broca's area: the inferior frontal gyrus, right pars triangularis (*t* = 5.72, p‐FDR=0.0033), and the right inferior frontal gyrus in the language network at [54, 28, 1] (*t* = 4.91, p‐FDR=0.0092). Note that none of the important regions for the DMN were included in the results of the ROI‐to‐ROI analysis for this schema‐match/schema‐mismatch contrast.

**TABLE 6:** Results of ROI‐to‐ROI Connectivity Analysis for Contrasts measured in the First USL Run between the Schema Matching and Mismatching Groups

Schema‐matching group versus schema‐mismatching group (in 1st USL)			
Seed/Sources	Targets	T	p‐FDR
networks. Language.pSTG (L)(−57, 47, 15)	atlas. PostCG l	5.62	0.004
	atlas.CO l	4.93	0.0056
	atlas.PO l	4.87	0.0056
	networks. SensoriMotor. Lateral (L) (−55, −12, 29)	4.82	0.0056
networks. SensoriMotor. Lateral (L)(−55, −12, 29)	networks. Cerebellar. Posterior (0, −79, −32)	5.58	0.0044
networks. Cerebellar. Posterior (0, −79, −32)	networks. SensoriMotor. Lateral(L)(−55, −12, 29)	5.58	0.0044
	atlas.CO l	4.88	0.0089
	atlas.PT l	4.74	0.0089
atlas.IFG tri r	atlas. Cereb8 r	5.72	0.0033
atlas. PostCG l	networks. Language.pSTG (L)(−57, 47, 15)	5.62	0.004
atlas.CO l	networks. Language.pSTG (L)(−57, 47, 15)	4.93	0.0098
	networks. Cerebellar. Posterior (0, −79, −32)	4.88	0.0098
atlas. Cereb2 r	networks. Salience.AInsula (R) (47, 14, 0)	5.27	0.0084
atlas. Cereb8 r	atlas.IFG tri r	5.72	0.0033
	network. Language.IFG (R) (54, 28, 1)	4.91	0.0092
networks. Salience.AInsula (R) (47, 14, 0)	atlas. Cereb2 r	5.27	0.0084
atlas.AG l	networks. FrontoParietal.LPFC (L) (−43, 33, 28)	−5.59	0.0043
networks. FrontoParietal.LPFC (L) (−43, 33, 28)	atlas.AG l	−5.59	0.0043

## 4 DISCUSSION

### 4.1 Connectivity changes across the switch in learning methodology

We examined three types of t‐contrasts between the ROI‐to‐ROI FC values brought from the listening portions of the first USL, SL composed of three runs, and the final USL that was identical to the first one. The FC toolbox (CONN) enables statistical evaluation of all combinations across 132 areas and 32 representative nodes with MNI coordinates of the eight intrinsic networks. ROI‐to‐ROI contrast analysis, although on a rough scale for a brain map, would be the most effective exploratory approach to the neural basis of *meta‐*concepts, such as USL or SL frames, which are either too general and abstract or too large‐scaled to narrow down and underpin any voxel‐wise signal interpretation that would be supported in the existing literature. The ROI‐to‐ROI connectivity analysis was a provisional way of evaluating the task‐state FC that was expressed simply as an amalgam of genuinely task‐evoked active and hodological interactive effects; the correlation computed in the default manner was considerably inflated by that mixture so it does not represent the exact amount of interregional functional synchronization (Cole et al., 2018; Janata et al., 2002). However, we assessed the first‐order effects of task‐evoked activations as well as FC effects and used this confounding coefficient as the neural “representation” of a schema for methodologically oriented language learning.

The first contrast, set between the first and second USL runs, represents exclusively the order effect that the medial SL runs engendered as an interference modulator that acted on the identical USL content (Table 4). The traditional language network between the left inferior frontal gyrus, pars opercularis, and the left posterior middle temporal gyrus was detected as a crucial edge where the association was significantly stronger in the first USL than in the second one. This result is convincing if we consider the posterior effects of SL that could interfere with spontaneous linguistic performance in some subjects, that is, efforts to find correct answers independent of their original propensity for sense in language. The other implications of this contrast would be related to the efficiency of the short‐term working memory in the first USL run, given that fresh exposure to a sound stream might activate the liveliest response in the subject's task of segmentation and retaining knowledge. Price (2012) claimed that recruitment of the left inferior frontal and temporo‐parietal areas during auditory segmentation tasks is a consequence of auditory short‐term memory. In this research, selective involvement of the left inferior frontal gyrus can be ascribed to the role of this area in sound segmentation and maintenance of auditory working memory (Burton et al., 2000; Hsieh et al., 2001; Pedersen et al., 2000; Poldrack et al., 2001). Regarding the significant association between the left inferior frontal gyrus and the left amygdala, the argument that is noteworthy is the theory of mediation between affect and cognition, especially concerning the formation of memories in acquisition of a second language (DeLuca et al., 2019). In this respect, it has been acknowledged that the amygdala is identified as a hub of the corticofugal path of memory formation that is responsible for regulating and appraising the objects to be learned and for maintaining sufficient motivation in learning (Schumann, 1990, 2001). The USL‐related temporal contrast was thus emphasized by the quality difference in performing the task, and it is possible that the SL altered the USL with the same task contents and conditions.

Interestingly, there is no information on this contrast pertaining to the DMN or its reorganization process, at least at the level of the subjects overall. The DMN was featured through the second contrast, which was between the first USL and the following SL runs. Compellingly, as indicated in Table 4, the DMN, and in particular its posterior subsystem, subserves the USL in the genuine condition and without confounds from the traces of memory training of SL. Compared with SL, the within‐connection during the first USL was extremely strong because it encompassed the bilateral inferior parietal lobules and the medial nodes of the posterior cingulate cortex and precuneus. Presumably, some inward thoughts and a flash of intuition for choosing parcels of sounds as meaningful to the subject activated the DMN during spontaneous activity in USL. It is likely that the DMN plays an important role in USL as long as the effects of SL are filtered to leave the DMN as the marker of pure USL. In contrast, the neural circuit of SL was identified, by force of contrast, in the edges between the posterior part of the left superior temporal gyrus (Wernicke's area) and the juxtapositional lobules bilaterally. According to recent studies, the former corresponds to the auditory modality aspect of language processing while the latter is also specified for relevant functions, especially inner speech mechanisms during language encoding, lexical disambiguation, syntax, and prosody integration, (Hertrich et al., 2016; Saur et al., 2010). We will underscore the importance of this connection later in the between‐subject analysis as a clue to the success of updating “schema” or “tuning” in the context of Rumelhart and Norman's theory of learning. In this section, we provide the outline for neural underpinning of the SL task in contrast with the functions of the DMN as a symbol of genuine USL.

The third contrast assigned between the second USL and the preceding SL runs was characterized by a set of multimodal between‐ROI edges featured in the former but not in the latter. The connectivity between Heschl's gyri (auditory cortex) and the lingual gyri (visual cortex) that increased in the second USL run when compared with the SL runs might reflect the auditory occipital association (AOA). The AOA, as a synchronization between the different perceptive modalities, represents, according to Cate et al., the persistent engagement of auditory attention; we can recognize this phenomenon when boosted in more difficult listening conditions (Cate et al., 2009) that need additional support from the visual cortex. Unexpectedly, this intriguing phenomenon emerged in the second USL run, which should have been more relaxed in the sense that only the subject's spontaneous reactions were required. A possible explanation for this finding is that the second USL run was affected by the executive control needed in the preceding SL, such that the subjects were no longer relaxed and suspected hidden correct answers. The neural mechanism underlying AOA remains unclear, in particular very little is known about the occipital regions (particularly the lingual gyrus) engaged in AOA and their specific functions with respect to auditory processing (Burton et al., 2005; Hasegawa et al., 2002; Janata et al., 2002; Zimmer & Macaluso, 2005). The remaining hubs in the third contrast other than those that were modality‐specific were the bilateral superior parietal lobules, which were connected to the right inferior frontal gyrus (the homologous portion of Broca's area) or the left anterior insula, representing the SN (Figure 4). The left superior parietal lobule is known to be a central region in the ECN involved in working memory‐related task performance (Koenigs et al., 2009; Liang et al., 2016). Interestingly, increased connectivity between the left superior parietal lobule and the right inferior frontal gyrus has been cited previously as evidence of brain plasticity promoted after 4 months of learning a second language (Bubbico et al., 2019). Furthermore, accumulating evidence indicates that the right inferior frontal gyrus plays a role in inhibiting control of word choice in language (Abutalebi & Green, 2016; Aron et al., 2004). We can interpret the SL‐ECN link that connects the left anterior insula with the right superior parietal lobule in light of the resource allocation theory of Lerman et al. (2014), who posited that the SN simultaneously influences the DMN and ECN and balances the allocation of resources for bidirectional control of cognitive activity. This balance has been identified to be disrupted in individuals with Internet gaming disorder (Zhang et al., 2017). Note that the dominant DMN of the first USL minus the SL runs again involves a portion of the SN that presumably influences the DMN and reduces the activity of the ECN, which connects the anterior cingulate gyrus and the parietal operculum.

### 4.2 Skilled learners coactivate bihemispheric auditory language resources with the DMN

The analyses to this point were performed by including all the subjects regardless of their individual variability in performance (in SL) and preference (in USL). From this point onward, we treat the between‐subject contrasts based on these two viewpoints. The difference in precision of SL performance (between the high‐ and low‐score groups) became manifest in FC only after finishing all the tests in the SL session, that is, during the final USL run when the responses were made spontaneously (Table 5). The interregional interactions were expanded to the bilateral areas, which were homologous in structure and to some extent in function for natural linguistic processing (left superior temporal gyrus) and complex sound or spectrotemporal pattern analysis (right planum temporale) (Griffiths & Warren, 2002; Obleser et al., 2008). Although pitch processing and assimilation of experience with tones (Xu et al., 2006) are often ascribed to the left planum temporale, the contralateral hemisphere is considered to play a role by connecting filtered acoustic information to areas of higher‐order cognitive function (Griffiths & Warren, 2002; Price, 2006). We noticed that during FC in the high‐score group, the right hemispheric nodes were located at the corresponding part of the left temporal language network, so were putatively concerned with speech recognition in parallel with Wernicke's area. Furthermore, the left posterior temporal lobe as a hub in this skilled learner characteristic connection was linked to the posterior subsystem of the DMN (precuneus and posterior cingulate cortex). This suggests that the group of higher scorers did not have to mobilize task‐positive resources to maintain a relaxed state with a moderate cognitive burden (Pyka et al., 2009) and spontaneous reactions in the final USL run. As already mentioned, many DMN associations emerged in the first (but not the final) USL when contrasted with the SL conditions. This long‐lasting participation of the DMN might allow us to isolate its significance in the success of USL and SL hybrid learning.

### 4.3 Difference in quality of learning between schema‐matching and schema‐mismatching groups

In this section, we analyze the reason why and how the schema‐matching and schema‐mismatching groups diverged in the course of switching learning methodology. The distinction between these groups involves intricate phases that originate from the complexity of the artificial grammar languages used in this experiment. The schema‐matching group, which more or less showed the S‐HiLo‐biased decision, was unarguably assisted by favorable prosodic features that were consistent across listening and evaluation in USL. The schema‐mismatching group followed the S‐LoHi order, which was unfavorable for consolidating word identity; for this syntactic pattern, pronunciation differed between speech and recognition of words. Interestingly, no subject in the schema‐matching group made any comment on the post‐hoc questionnaire about syntactic information, although they might have found that condition favorable. One explanatory hypothesis is that they were inclined toward a type of pitch learning where congruity of the stress position in the USL material could provide a key to word segmentation. However, viewed in light of the responses to SL testing, they did not persist in using prosodic cues exclusively but succeeded in “tuning” their preference schema, which could reflect not only aspects of prosodic learning but also sequential learning of lexemes independently of auditory tone. In contrast, the schema‐mismatching group that never showed a preference for S‐HiLo might have had incongruity in the stress positions of the S‐LoHi words between the two steps in each SL run. If they adhered nevertheless to the S‐LoHi pattern, discarding the auditory fluctuation between the P‐LoHi in speech and the P‐HiLo in the test, their linguistic schema may have remained “accreted” to the syntactic formation built on the arrangement of frequent and nonfrequent syllables.

Interestingly, the difference in performance quality between the schema‐matching and the schema‐mismatching groups is in line with the results of a comparison of implicit (not rule‐instructed) and explicit (rule application) learning processes when performing a probabilistic continuous fine motor task (Green & Flowers, 2003). The schema‐matching group could benefit from the conformity of its horizon of expectations (private proto‐schema) with hidden rules that could tacitly lead the learning responses to a new control frame for adaptation. Similarly, implicit and unconscious acquisition of skills, when successful, is often exempted from additional cognitive demands, given that under explicit learning conditions, costs that would interfere with learning might arise when mismatch of schemas triggers a supplemental search for unknown rules that are unexpected but unambiguously acceptable. These decrements in learning performance during SL afford a valuable insight into how we assure favorableness of USL, that is, the original inclination of individuals toward a set of patterns that would be revealed ex post facto as representation of rules. For better learning, we should take into consideration the role of each subject's proto‐schema, which is predictable from his/her original response pattern. When this situational condition is fulfilled for a group of better performers through latent schema‐matching in the USL, we can measure the effects through the enhanced connectivity between the DMN and the other task‐related intrinsic networks. In this study, we found that the DMN characterizes the first stage of USL, but not SL (regardless of performance quality), and that the final achievement (for successful subjects) of whole learning in concordance with the task‐related (in our case, auditory‐weighted linguistic) networks (Figures 2 and 3).

However, the DMN did not represent the disparity in preference during the initial USL run (it was not included in the ROI‐to‐ROI contrast map of connectivity between the groups; Table 6; Figure 4), although at this stage the schema‐matching group could already proceed smoothly with learning by receiving favors for existence of disyllabic words, which were preferred in terms of both syntactic and prosodic HiLo patterns. In contrast, subjects in the schema‐mismatching group might have suspected that there was a dilemma regarding the choice because the syntactic LoHi pattern they preferred was implemented in disyllabic units with the prosodic LoHi pattern that they did not always favor. Moreover, the stress position in the syntactic LoHi pattern words was inverted in the testing phase of the USL run, which could be perplexing for those who consider AG rules owing to the lack of prosodic consistency. In the first spontaneous learning session, such complexity in the preference retrieval process was not reflected in the activity of the DMN; rather, it was the neural basis of the “self‐supervised learning” aspect (Kröger & Bekolay, 2019) that could capture the trace of the schema matching through the input from exposure to the speech stream. It may be that this “self‐supervised learning” has something to do with “superlearning” (Caligiore et al., 2019) as an integration of SL, USL, and reinforcement learning. “Superlearning” implies a set of learning mechanisms that act in synergy to trigger widespread neuro‐transformation at various modality and abstraction levels that are transmitted through multiple cortical/subcortical pathways and loops, including the cerebellum.

Self‐supervised learning‐recruited networks that started at the peripheral nodes in the cerebellum bilaterally encompassed crucial regions for cognitive management in linguistic processing, for example, the right insula representing the SN, the right homologous portion of Broca's area that is involved in linguistic executive control, and some left lateral regions included in the sensorimotor network. This finding is consistent with the suggestion of Caligiore et al., (2019) that USL mechanisms could boost cerebellar performance during SL and for the schema‐matching group already in the opening stages of USL. Moreover, the argument regarding cerebellar learning mechanisms can be validated by referring to the many papers on the cognitive functions of the cerebellum as well as labeling of the Connectivity toolbox for the cerebellar subsystems. For example, there has been mounting interest in the involvement of Crus I and I in the cerebellum in lexical acquisition and word retrieval based on their roles in executive control, salience detection, episodic memory, and self‐reflection (Habas et al., 2009). The functional topography of the cerebellar cortex consists of a set of complicated enclaves that do not conform to the geographic features of the subregions but instead reflect the motor and cognitive functions to be projected to the FC in the forebrain (Guell et al., 2018; Sokolov et al., 2017). According to Buckner et al. (2011), approximately half of the cerebellum, especially Crus I and II (language areas in the cerebellum), is functionally connected to the cortical regions related to the DMN (Dobromyslin et al., 2012). For the neural response of schema‐matching subjects, recruitment of the cognitive (and linguistic) cerebellum will be assessed by virtue of how the DMN represents success in USL (Figure 4).

### 4.4 Limitations and extensions

This study has some limitations, some of which may merit future inquiry. We attempted to analyze the characteristics of the subjects in light of three stages of changes in the schema that Rumelhart and Norman theorized could explain the evolution of learning, namely, “accretion,” “tuning,” and “reconstruction.” In our study, possibly because of the small cohort size for scans, we could not record any dynamic sample of the final stage (“reconstruction”), which should have created a new schema for fundamental renovation of the learning strategies. There were no cases of schema‐mismatching whereby a subject with an unfavorable proto‐schema during the SL runs went on to have results accurate enough to warrant reclassification in the schema‐matching group.

In addition to the paucity of subjects, it was difficult to include scenario‐making or contextual simulation for reconstruction of schemas in the experimental design because we had to allow for timing of critical triggers for shifting of the schema while controlling for any spurious order effects. The pitch control of the speech stream was unavoidably imperfect for the three artificial speeches (sound streams) in the SL runs because the Macintosh specifications of the “say” command aim at synthesizing voices that have the most natural pitch pattern for the given natural languages. This factor gave rise to a slightly complicated material structure that could confuse subjects and worsen their performance. These trivial aspects of experimental stimulation and the research design of alternating the USL and SL for detection/retrieval of similar knowledge targets would not guarantee generalizability of the current approach to mixed learning methodologies or have neural correlates that could be mapped to “schema” dynamics. Moreover, in terms of more in‐depth analysis of FC, there are still aspects of our experimental design that are yet to be uncovered. Given that the whole session was composed of a variety of stimuli and tasks that took considerable time for repeated learning and testing, we could not set any independent resting state for fMRI that was sufficiently long, stable, and immune to fluctuation for more than several minutes. Such scan data should have enabled a total independent component analysis (ICA) to determine, in a bottom‐up manner, the intrinsic functional networks that would be specific to the experimental groups. This is the reason why we opted to use the network templates prepared in CONN in a top‐down manner to perform the connectivity analysis.

## 5 CONCLUSION

Despite these limitations, our study demonstrated the dynamic topography of enhanced or weakened connectivity and has helped to elucidate the neural basis of schema modulation in learning development. Notably, the inquiry of functional brain mapping for abstract and comprehensive (meta‐) concepts like USL, SL, and hybrid superlearning should be started from re‐acknowledgement of global activation patterns across brain regions with fluctuation between canonical intrinsic networks serving as FC templates. In that sense, the contribution of the present study to neuro‐pedagogy is that we determined the synchronized neural responses of ROIs under different learning condition and the order effect. The results of the current ROI‐to‐ROI analysis for contrasting learning conditions, although inevitably coarse, pave the way for more detailed voxel‐based investigations using other experimental materials and methods for disentangling neural schema factors. Involvement of DMN in genuine USL engages the cerebellum in self‐supervised learning, which can be called superlearning, and intervention of the SN for switching the learning methods by adjusting resource allocation to the DMN and the executive control network in turn, are all functional networks and their alternation will be reconfirmed by further empirical support. A future study can expand beyond the present research paradigm but should be in keeping with the source ROIs found to underlie the dynamic process of language acquisition.

## CONFLICTS OF INTEREST

The authors declare no conflicts of interest.

### PEER REVIEW

The peer review history for this article is available at https://publons.com/publon/10.1002/brb3.2157.

## Supporting information

Supplementary Material

## Data Availability

Data available on request from the authors.
