# Supplementary material for: Task‐induced brain functional connectivity as a representation of schema for mediating unsupervised and supervised learning dynamics in language acquisition
Source: Brain Behav. 2021 May 5;11(6):e02157. doi: 10.1002/brb3.2157 (PMC8213930; doi:10.1002/brb3.2157)
Supplement: Supplementary file 1 — Supplementary Material [file BRB3-11-e02157-s001.docx]

Supplementary Information

1. Seed-to-ROI analysis

**MATERIALS AND METHODS**

**Methodology**

Seed-to-region of interest (ROI)-to-ROI FC maps were created in both the first-level and second-level connectivity analyses. We applied seed-to-voxel analyses to several ROIs that might be key hub regions in some (presumably canonical) connectivity patterns, based on their systematic edge contrast features.

**RESULTS**

**Contrast analysis between the first USL, SL, and second USL runs**

Given that we initially hypothesized the importance of the DMN in neural processing during USL, we ran a seed-to-voxel analysis for this contrast, setting the seed ROI to the posterior cingulate cortex in the DMN at [1, -61, 38] close to the precuneus. Figure A shows four significant clusters under the conditions of the height threshold at p<0.05 FDR and the peak voxel threshold at p<0.05 FWE, which were represented by the MNI coordinates of [-10, -52, +20], [-46, -68, +20], [-16, -30, +6], and [+10, -46, -50] (the sizes were all p<0.05, family wise error [FWE]), mainly encompassing i) the precuneus and posterior cingulate gyrus, ii) superior division of the left lateral occipital cortex, and left angular gyrus, iii) bilateral thalami, and iv) right cerebellum 9, respectively. It is noteworthy that all the clusters except iii) covered the posterior system of the DMN in the brain.


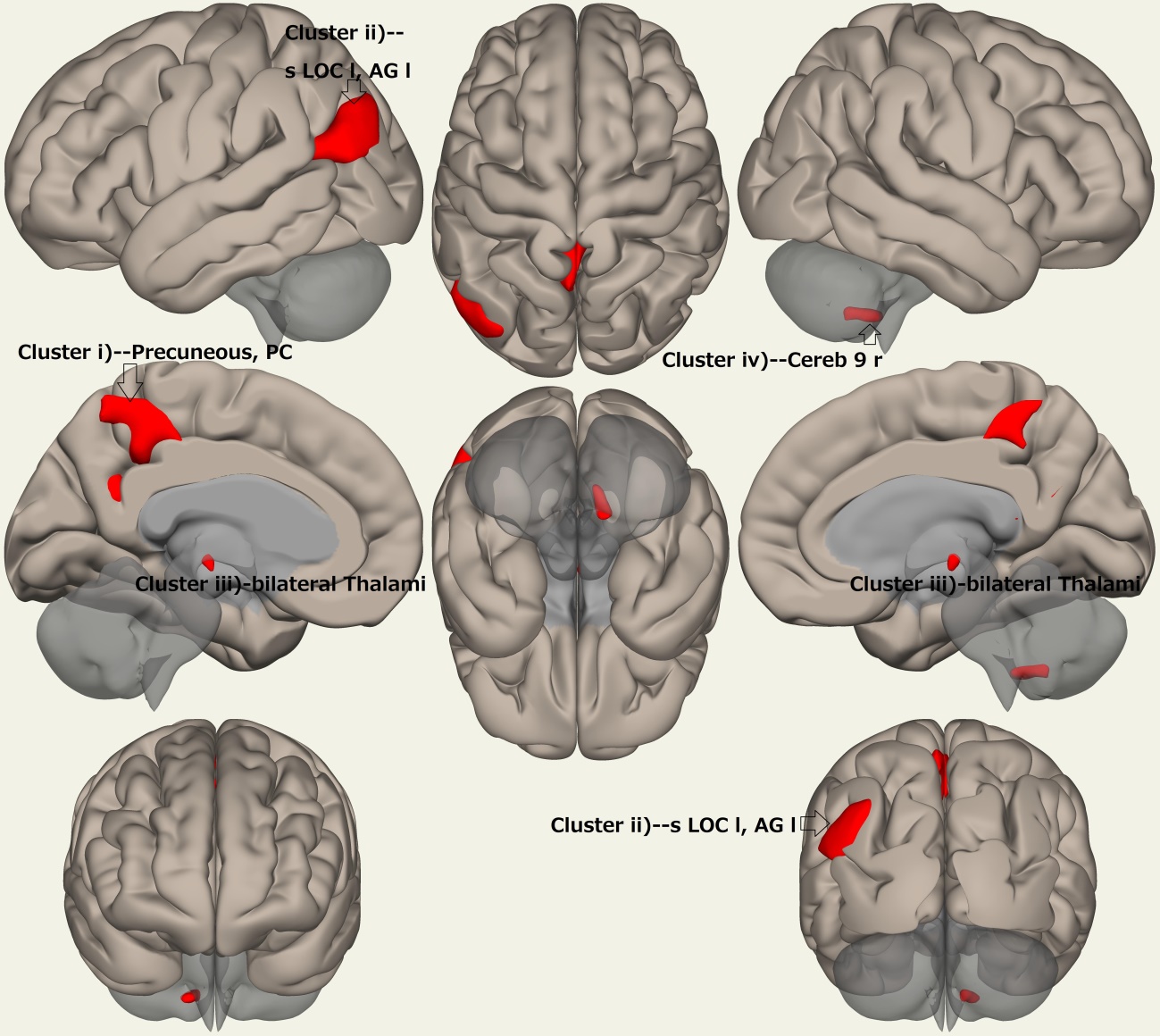


Figure A. Seed-to-voxel analysis for contrast between the first USL and the SL (first USL > SL) revealed three DMN-related clusters (posterior cingulate cortex/precuneus, left angular gyrus l, and right cerebellum 9) extracted by setting the source region of interest at the precuneus. DMN, default mode network; SL, supervised learning; USL, unsupervised learning

**High score group vs low score group**

For a further investigation into the neural underpinning of linguistic USL, we selected the hub of an ROI-to-ROI-based significant subnetwork (Figure 2 in the main document), i.e., the posterior portion of the left superior temporal gyrus (Wernicke’s area) as a seed for seed-to-voxel analysis of the contrast between the high and low score groups in the final USL at the end. The Figure B elicits two significant clusters under the conditions of the uncorrected height threshold (p<0.0005) and the corrected cluster size threshold (p<0.05) using FWE. The first cluster has a peak at [+58, -50, +10] (MNI coordinates) and a size of 336 voxels (p=0.000264, FWE) covering the right temporo-occipital portion of the middle temporal gyrus and the right angular gyrus as a crucial node of the DMN. The second cluster records a peak at [-8, -68, 34] and a size of 121 voxels (p=0.047744, FWE) covering the precuneus, which represents the medial system of the DMN. This result emphasizes the important role played by the DMN in successful performance of skilled learners obtained by accumulation of USL of language through listening exercises.


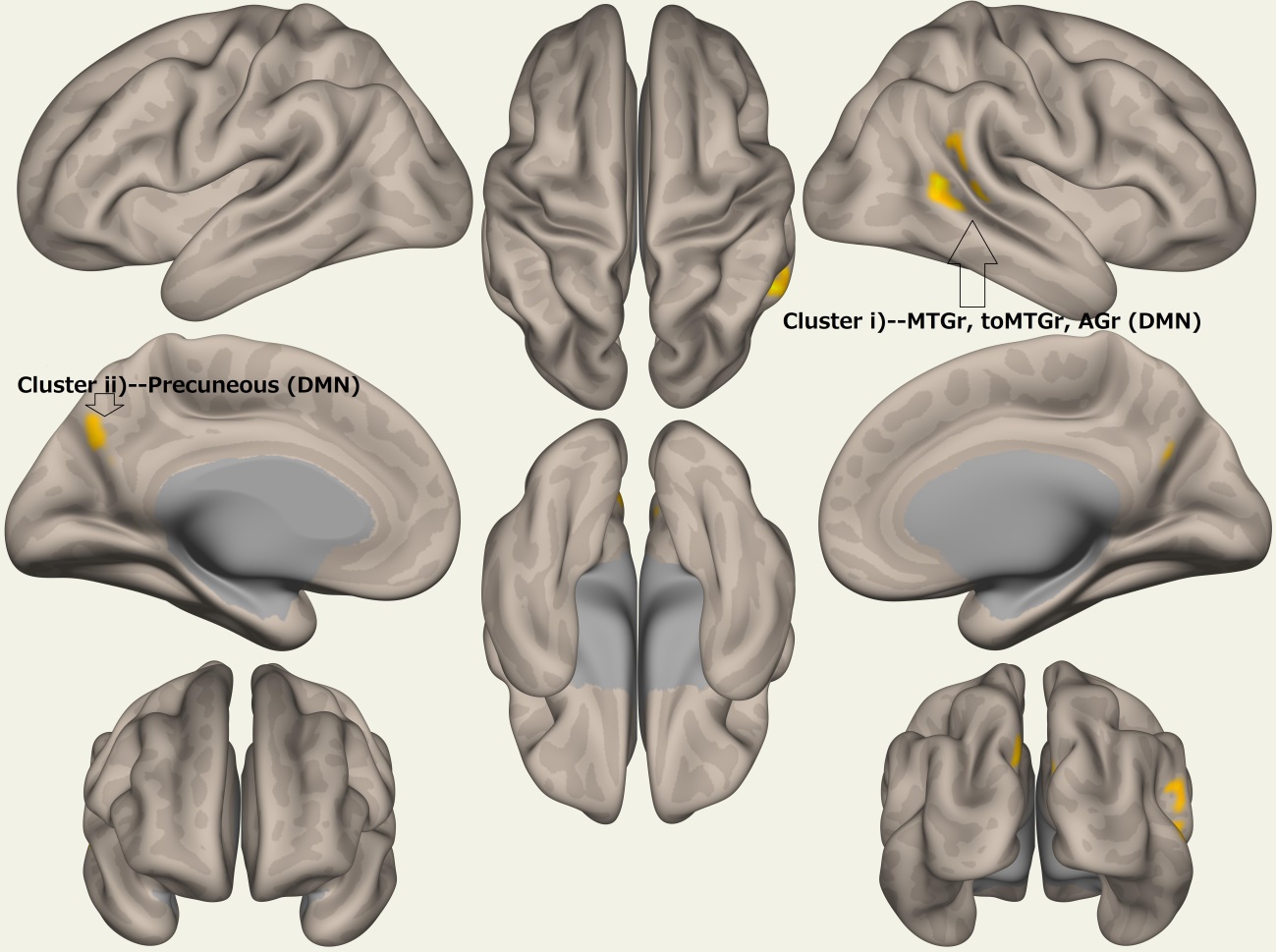


Figure B. Z-score mapping result of the seed-to-voxel analysis setting posterior portion of the left superior temporal gyrus (Wernicke’s area) as the seed. A temporal-parietal subsystem of the dorsal posterior default mode network was detected as a significant cluster for this seed. ROI, region of interest; SL, supervised learning; USL, unsupervised learning.

**Schema-matching group vs schema-mismatching group**

With a view to tapping into the voxel space of targets, we paid attention to the node of the highest degree in this ROI-to-ROI-based significant subnetwork (Figure 4 in the main text), i.e., the posterior cerebellar networks featured by the developers of the CONN in the seed list with the center coordinate at [0, -79, -32], as a seed of seed-to-voxel analysis for the schema-matching/mismatching group contrast in the first USL. A single cluster was recorded (Figure C, above) as significant under the conditions of a height threshold of p<0.05 FDR and cluster size threshold of p<0.05 corrected with a peak at [-56, -10, +6] (MNI coordinates) and a size of 199 voxels (p=0.000001, FWE) covering some parts of the left central opercular cortex, left planum temporale, left planum polare, and left Heschl's gyrus, which are regions involved in attentive listening. This cluster could include regions that are important for control of executive function in linguistic performance, such as the anterior division of the left superior temporal gyrus, left postcentral gyrus, left planum polare, left frontal operculum cortex, and posterior portion of the left superior temporal gyrus under the more relaxed height condition of p<0.001 uncorrected and the same cluster size condition of p<0.05 FWE. Interestingly, if we selected the left insular cortex in the SN as a seed, we obtained two significant clusters (a height threshold of p<0.001 uncorrected and a cluster size threshold of p<0.05 FDR), the first one represented by [+38, -74, -28] and encompassing the right cerebellum crus I and II and the second [-38, +14, +54] including the left middle frontal gyrus. These results suggest that the subjects who could easily “tune” the schema for learning as a result of their preference for a S-HiLo pattern activated from the beginning of the functional intrinsic networks where the full-fledged engagement of the cerebellum could be confirmed (Figure C, Below).


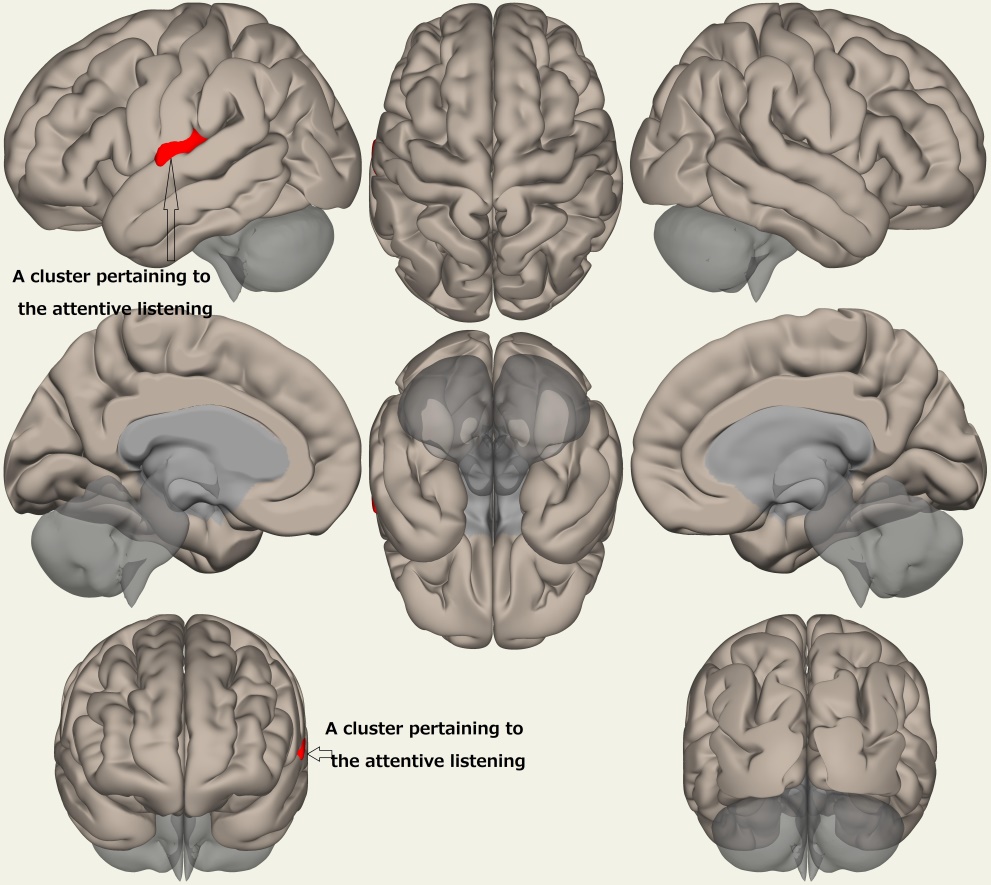

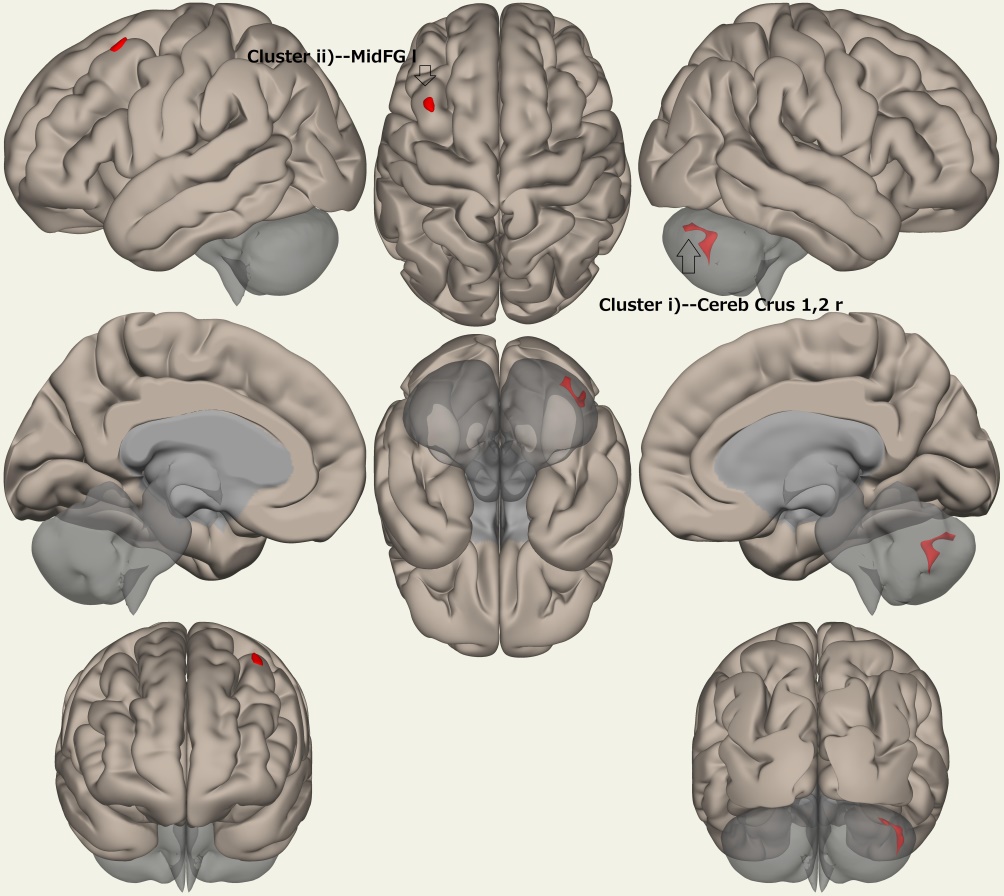


Figure C (a). Above: Z-score mapping result of the seed-to-voxel analysis setting the posterior cerebellar networks as the seed. An auditory network for attentive listening was selected as a significant cluster for the seed of the posterior cerebellum. Figure C-(b). Below: Two clusters in the right cerebellar lobules 1 and 2 and the left middle frontal gyrus were obtained from the seed-to-voxel analysis setting as the seed of the left insular cortex representing the salience network. IFG, inferior frontal gyrus; pSTG, posterior portion of the superior temporal gyrus; ROI, region of interest; SL, supervised learning; USL, unsupervised learning

**Smooth schema tuners or accreted schema holders?**

There remain important concerns pertaining to the disparity in proto-schema (or preference-based selection of segments), which is viewed according to the three-tier schema evolution theory of Rumelhart and Norman. If we believe that the first USL run allows the opportunity for self-supervised (super) learning (Caligiore et al., 2019; Kröger & Bekolay, 2019), subjects who participated in this rich process could accumulate partial information in the same vein by appropriate exposure to learning materials. This consolidation step corresponds to what Rumelhart et al. defined as “accretion”; however, the schema-mismatching subjects had the same knowledge and also took part in the acquisition process to reinforce their proto-schema, which stressed only one type of acquisition cue (in this case, syntactic LoHi pattern) by suppressing the other (a contradictory prosodic cue). Therefore, regardless of whether or not schema matching occurred, the activity of the DMN prior to SL could be defined as “accretion”, in the sense of “learning by adding new data structures to the existing data base of memory” (Rumelhart & Norman, 1976). Thus, the DMN represents a proto-schema as a neural response pattern for eliciting each individual’s spontaneous preference. It becomes conceals and remains latent (even more robust) posterior to the intervention of teachers in the SL runs.

The next modulation of the schema, called “tuning”, hinges on the disparity between subjects that become salient during SL runs. The phase-in of minor schema changes is named “tuning” by Rumelhart et al. and can be assessed by the success or failure of memory retrieval during the testing procedure on SL runs. Since the 36-segment word candidates are disyllabic patterns consisting of P-HiLo (so pitch weights are the same as in the testing), P-LoHi (so the stress position was changed), and prosodically neutral patterns (Table 1), at least partial updating of the proto-schema was mandatory in the three consecutive SL runs. The subjects showing schema mismatching failed to cope with instances where although the combination of phonemes was identical, the pitch pattern differed between the input (immersion) and output (memory retrieval). It is likely that as a consequence of this unsuccessful “tuning”, wherein the discordance between the two factors of the proto-schema interfered with memory retrieval of correct answers, these subjects were obliged to adhere to the “accretion” step and refuse to make any change in syntactical preference (to S-LoHi). We hypothesize that the degree to which "tuning" competences are uneven (across the schema matching and mismatching subjects) was related to the paucity of statistically significant between-ROI edges at which associative strengths were larger in the listening phases of the SL than those of the USL. Figure D shows the result for ROI-to-ROI connectivity analysis of the contrast between SL and the first USL (SL > first USL) for the schema-mismatching group only for the subject effect. There were two (negative edges) for the schema mismatching group under the condition of p-FDR <0.01: i) between the posterior portion of the left superior temporal gyrus and the right juxtapositional lobule (t=-5.64, p-FDR=0.0066) and between ii) the posterior part of the cerebellar networks at [0, -79, -32] and the left planum temporale (t=-5.46, p-FDR=0.0088).


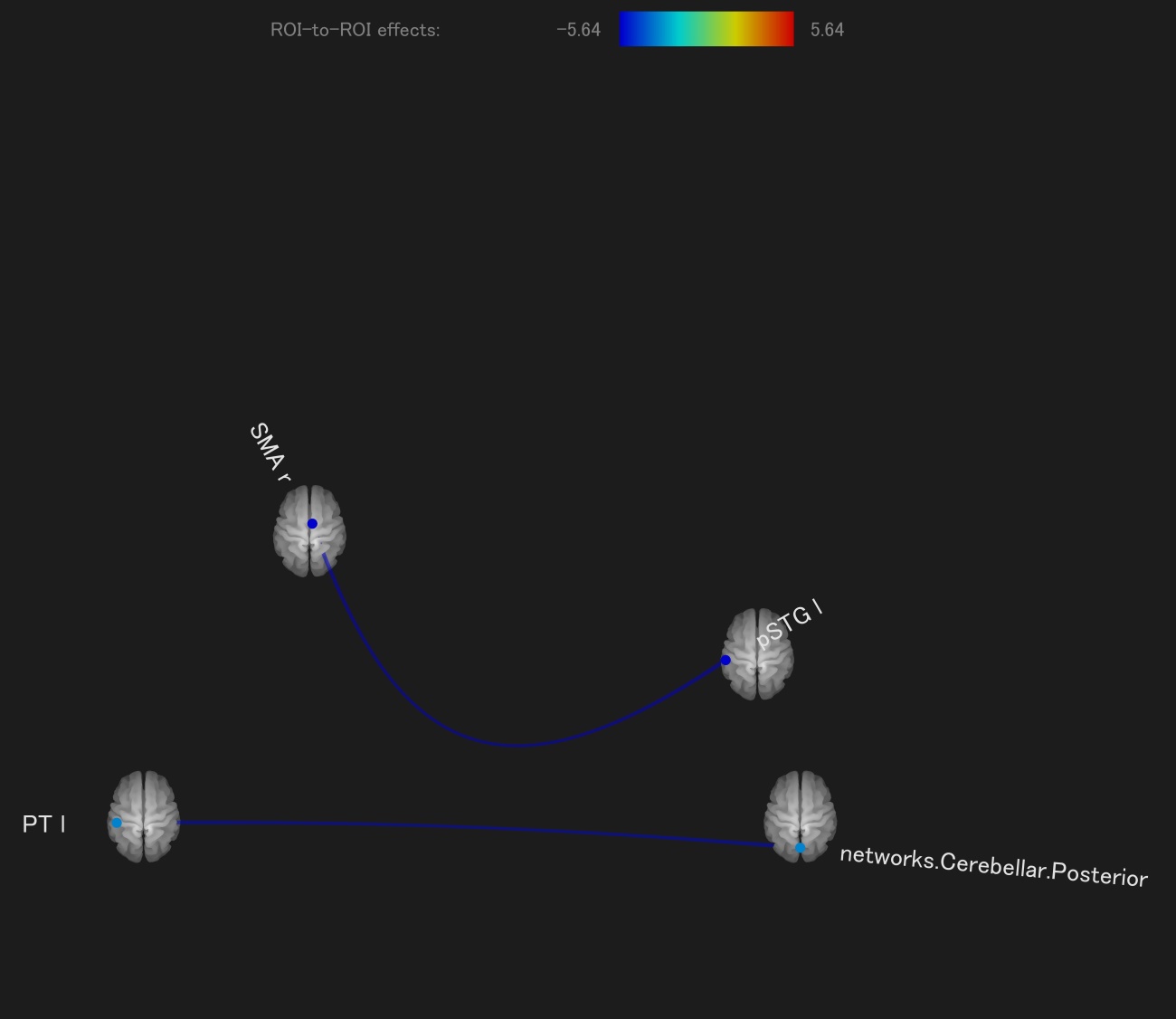


Figure D. Result for ROI-to-ROI connectivity analysis of contrast between the SL and the first USL (SL > first USL) for the schema-mismatching group. ROI, region of interest; SL, supervised learning; USL, unsupervised learning

Edge ii) appeared already in the contrast at the first USL run between the schema-matching and schema-mismatching groups (Table 6). Edge i) between the left superior temporal gyrus and right SMA, which is not featured in Table 6, might reflect executive control for “tuning” schema because it was the same as one of the ROI-to-ROI connections extracted from the contrast of SL versus the first USL (Table 4). It was negatively correlated with the activity of the DMN in the whole subject analysis. Hertrich et al. (2016) postulated that the SMA plays the role of coordinator between phonological-phonetic sequencing in the left hemisphere and timing of prosodic events in the right. Since this coordination holds true irrespective of linguistic constituent levels, such as syllable structure, syntax, and phrasing (Hertrich, Dietrich & Ackermann, 2016) we can speculate that the neural circuit of schema tuning includes the bilateral SMA as principal nodes in language learning.

Nevertheless, the finding of a striking disparity between smooth schema tuners and accreted schema holders is intriguing. Smooth schema tuners could perform the word segmentation task well by taking advantage of their syntactic preference and its fortuitous matching with prosodic information. In contrast, accreted schema holders adhered to their opposite syntactic preference, despite the prosodic order between learning and testing being reversed for their choice. Our experiment did not record any trace of “restructuring” schema because no one attempted to acquire entire priority over the pitch allocation to the transitional probability (S-HiLo) with the correct words. Few subjects converted their prediction patterns from S-LoHi to S-HiLo to improve their SL scores, so the divergence in schema updating was stopped at the stage of “tuning”, when the performance quality was contingent on the preference rate of the S-HiLo. The underlying cause of “tuning” failure might lie in the fact that the prosodic pattern of the key syllables for subjects preferring S-LoHi was systematically incongruent between listening (P-LoHi) and testing (P-HiLo) of the first USL. Neural representation of the schema “restructuring” remains an open question that should encourage a prospective study.

**Limitations and extensions**

A voxel-to-voxel analysis was set to the least restrictive independent component factor (No. 11: the best three matches to ICA_11 are language (r=0.338385) and salience (r=0.183219) with a kurtosis value of 4.8072 indicating a best match to language network and a second-best match to SN. Based on this IC, there was a cluster with a peak at [+00, -72, +24] in the precuneus (height threshold: p<0.001 uncorrected, cluster size threshold: p-FDR<0.05; Figure E for contrast in the high score group vs the low score group. Since ROI-to-ROI analysis detected a significant edge of the SN connecting the anterior cingulate gyrus and parietal operculum under the contrast between the first USL run and the SL runs, it may be that this cluster represents a part of the salience/ventral attention network (Schaefer et al., 2018; Yerys et al., 2018) and includes all the areas responsible for selective attention. Naturally, involvement of the precuneus arguably represents activation of DMN, so a new experiment should be planned and performed integrating an independent resting state and including more compact but equally effective tasks in a further fMRI study. We must address issues on the mechanism of FC switch in the areas where intrinsic networks show considerable overlap.


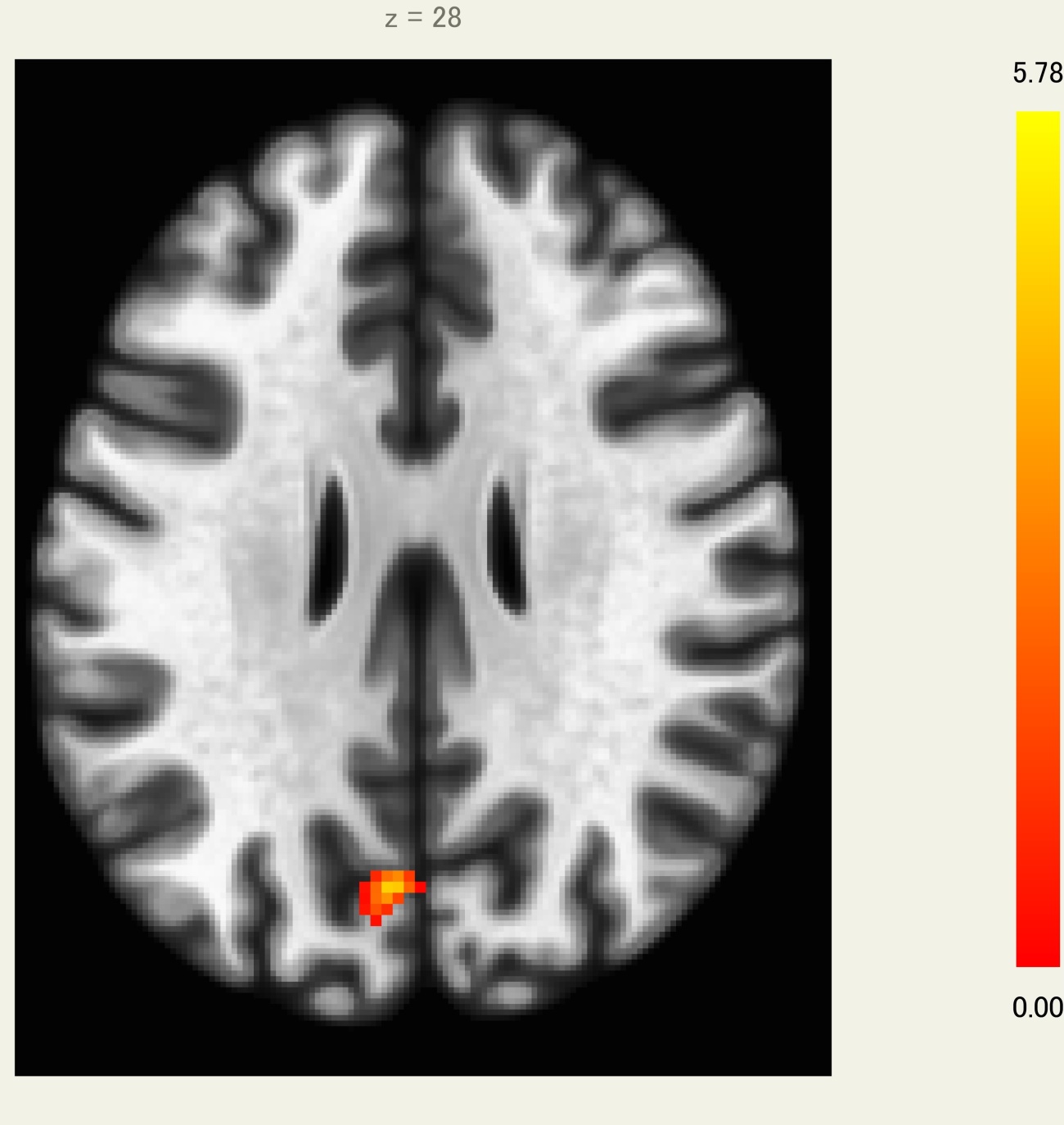


Figure E　Cluster in the precuneus extracted under the condition of high score versus low score from independent component matching with the template of language and salience networks (height threshold: p<0.001 uncorrected; cluster size threshold: p-FDR<0.05).

1. Supplementary Table
2. Supplementary Table

| SUBJID | Score Group  (High/  Low) | SL test records | USL1 Performance  Self-assessment (bad: 1/good: 0) | USL2 Performance  Self-assessment (good: 1/bad: 0) | USL  Understandability Self-assessment (4 points scale) |
| --- | --- | --- | --- | --- | --- |
| subject1 | 0 | 0.638889 | 1 | 1 | 2 |
| subject2 | 1 | 0.888889 | 0 | 0 | 4 |
| subject3 | 1 | 0.944444 | 1 | 1 | 3 |
| subject4 | 1 | 0.805556 | 0 | 0 | 3 |
| subject5 | 1 | 0.888889 | 1 | 0 | 2 |
| subject6 | 1 | 0.833333 | 1 | 1 | 3 |
| subject7 | 0 | 0.666667 | 1 | 0 | 2 |
| subject8 | 0 | 0.583333 | 0 | 0 | 2 |
| subject9 | 0 | 0.694444 | 0 | 0 | 2 |
| subject10 | 1 | 0.972222 | 0 | 0 | 3 |
| subject11 | 0 | 0.75 | 0 | 0 | 2 |
| sunject12 | 0 | 0.777778 | 1 | 1 | 2 |
| subject13 | 0 | 0.361111 | 0 | 0 | 2 |
| subject14 | 1 | 0.916667 | 1 | 1 | 2 |
| subject15 | 1 | 0.861111 | 0 | 0 | 4 |
| subject16 | 0 | 0.472222 | 0 | 0 | 2 |
| subject17 | 0 | 0.388889 | 1 | 1 | 2 |
| subject18 | 0 | 0.25 | 1 | 0 | 2 |
| subject19 | 1 | 0.916667 | 1 | 1 | 3 |
| subject20 | 1 | 0.805556 | 1 | 1 | 4 |
